# Network Signatures of IgG Immune Repertoires in Hepatitis B Associated Chronic Infection and Vaccination Responses

**DOI:** 10.1038/srep26556

**Published:** 2016-05-25

**Authors:** Ya-Hui Chang, Hui-Chung Kuan, T. C. Hsieh, K. H. Ma, Chung-Hsiang Yang, Wei-Bin Hsu, Shih-Feng Tsai, Anne Chao, Hong-Hsing Liu

**Affiliations:** 1 Institute of Molecular and Genomic Medicine, National Health Research Institutes, Zhunan 35053, Taiwan; 2 Pediatrics, En Chu Kong Hospital, Sanxia 23702, Taiwan; 3 Institute of Statistics, National Tsing Hua University, Hsin-Chu 30043, Taiwan

## Abstract

The repertoire of IgG antibody responses to infection and vaccination varies depending on the characteristics of the immunogen and the ability of the host to mount a protective immune response. Chronic hepatitis B virus (HBV) infections are marked by persistent infection and immune tolerance to vaccination. This disease offers a unique opportunity to discover key repertoire signatures during infection and in response to vaccination. Complementarity determining region 3 of an antibody heavy chain (CDR-H3) has a major impact on the antigenic specificity of an antibody. We used next-generation sequencing to characterize the CDR-H3 sequences in paired siblings of 4 families in which only one member of each pair had chronic HBV infection. Blood samples were obtained before and 2 weeks after HBV vaccination. The analysis revealed a huge network of sequence-related CDR-H3 clones found almost exclusively among carriers. In contrast, vaccination induced significant increases of CDR-H3 cluster diversities among siblings without hepatitis B. Several vaccination-associated clone clusters were identified. Similar findings of vaccination-associated clone networks were observed in healthy adults receiving HBV boosters. These strategies can be used to identify signatures of other infectious diseases and accelerate discoveries of antibody sequences with important biomedical implications.

The repertoire of IgG antibody responses to infection and vaccination varies depending on the nature of the immunogen and the ability of the host to mount a protective immune response. It is now known that there are huge variations in immune repertoires even between identical twins^1^. Sensitive and specific methods are needed to delineate these immune repertoires to better understand immune responses in the coming era of personalized medicine. People with chronic hepatitis B virus (HBV) infections are at high risk of developing hepatitis, hepatic cirrhosis and hepatocarcinoma^2^. Their clinical status and prognosis is currently defined by a variety of antibody responses. For example antibodies against core antigen (HBcAg) are hallmarks of past exposure to the virus^3^, and appearance of immunoglobulins against e-antigen (HBeAg) represents a stage shift for hepatitis B carriers^3^. Vaccines based on the s-antigen (HBsAg) are the most effective method to prevent chronic infections and associated liver diseases^4^. However, HBsAg vaccines are ineffective in HBV carriers because of virus-induced immune tolerance^5^. These distinct features of chronic HBV infections stimulated us to explore the IgG immune repertoire of HBV infections and response to immunization as a model to develop an immune repertoire-based approach to infection and vaccination.

Prior to the current era of next-generation sequencing (NGS)^6^ antibody responses could only be characterized at low resolutions by either cloning or spectratyping. Because nucleotides in the complementarity-determining region 3 of the heavy chain (CDR-H3) on most antibodies are sufficient to determine specificities^7^, sequence repertoires of this region can effectively serve as clone proxies of humoral immunity. Nucleotides flanking the CDR-H3 region are relatively constant and have been characterized with standardized numbering^8^. Appropriately designed PCR primers could adequately prepare CDR-H3-based immune repertoires for parallel sequencing. Consequently biological conditions can be defined in terms of immune repertoires at clonal resolutions. This helps to address questions from a numerical approach.

Many investigations adopting NGS-profiled B-cell immune repertoires have provided detailed insights in response to vaccination. For example the lineage structure of responding antibodies has been demonstrated for influenza vaccines^9^. A twin study revealed the stochastic or individual-specific effects on clone selections against acute antigenic stimuli from live-attenuated chickenpox^1^. A study involving multiple time points after HBV vaccination revealed sequence convergence mostly notable at 14 and 21 days later^10^. The dynamics of influenza vaccination were recently defined without the need for cell sorting^11^. Acute dengue fever was found to carry a convergent antibody signature^12^, but disturbances to immune repertoires from chronic infections remained elusive. A study of human immunodeficiency virus-1 (HIV-1) infections found skewed selections of antibody heavy chain families^13^. Finally, investigations of the repertoires of adults carrying cytomegalovirus (CMV) or Epstein-Barr virus (EBV) disclosed a few individualized phylogenetic trees without clear associations with either virus^14^.

In the current study we used next-generation sequencing to characterize the CDR-H3 sequences in paired siblings of 4 families in which only one member of each pair had chronic HBV infection. Blood samples were obtained before and 2 weeks after HBV vaccination. Analyses were performed with abundance-weighted heuristics of clonal transcripts under the assumption that amounts of clonal transcripts positively correlate with functional dominance of corresponding cells. For example plasma cells have very high amounts of clonal transcripts^15^ and numbers of related memory cells increase nearly to a hundred fold after vaccination^10^, both of which can be essentially described with abundance-weighted heuristics of clonal transcripts. The analysis revealed a huge network of sequence-related CDR-H3 clones found almost exclusively among carriers. In contrast, vaccination induced significant increases of CDR-H3 cluster diversities among siblings without hepatitis B. Several vaccination-associated clone clusters were identified. Similar findings of vaccination-associated clone networks were observed in healthy adults receiving HBV boosters.

## Results

### Studies of sibling pairs with and without chronic hepatitis B and response to vaccination

Four pairs of children born to HBeAg positive mothers were recruited from four different families (Table 1). In each family one child was an HBeAg carrier while the other was an HBsAg negative non-carrier. Liver-related serum transaminases were normal in both. The last dose of HBV vaccine had been administered at least two years after they were vaccinated at 6 months old as part of the national vaccination program. Blood samples were collected immediately before and 2 weeks after vaccination (Supplementary Fig. S1A). Anti-HBs antibodies for non-carriers were positive at either 6-month to 1-year of follow-up (family 1, 2, and 4) or before vaccination (family 3). Anti-HBs antibodies were not present in carriers as expected^5^. IgG repertoires of CDR-H3 were prepared from reverse-transcribed total RNA plus 2 runs of optimized PCR (Supplementary Fig. S1B and Supplementary Table 1). The underlying logic is that there is a positive correlation between the amounts of clonal transcripts and functional dominance of corresponding cells^15^. Examples include plasma cells that contain very high amounts of clonal transcripts^15^ and functionally related memory cells whose numbers can increase up to a hundred fold after vaccination^10^. Both conditions can be conceptually unified in terms of counts of related clones, or abundance-weighted heuristics. Raw reads from NextSeq 500 (Illumina, USA) were processed with both open-domain utilities and homemade Python scripts (Supplementary Fig. S1C). CDR-H3 in amino acids based on IMGT definitions^8^ was extracted for downstream analyses. Throughputs per sample in average were 2.2 ± 0.5 (SD) million reads belonging to 155.7 ± 42.5 (SD) thousand unique CDR-H3 clones (Table 1).

### Immune repertoires exhibited similar length distributions and amino acid compositions but could be distinguished from each other

**Figure 1:**
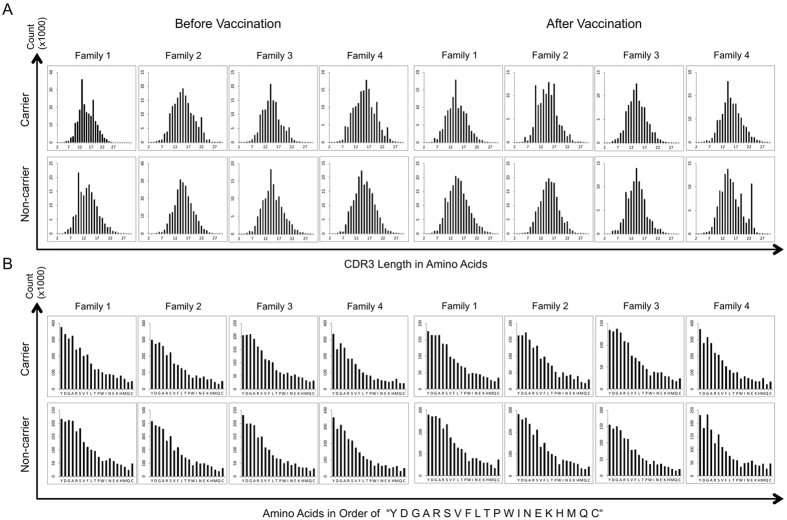
Distributions of CDR-H3 lengths and amino acid compositions. (**A**) CDR-H3 lengths were distributed as bell shaped curves regardless of HBV infection or vaccination. Mean lengths for both carrier and non-carrier children were identical at 13.2 ± 0.0 (SE) amino acids. (**B**) Composition of the amino acids resembled each other regardless of the clinical condition or vaccination. The most frequent amino acids in all samples were tyrosine, asparagine, glycine, alanine, arginine, and serine.

Lengths of IgG CDR-H3 immune repertoires were similarly distributed for all children in a bell shape manner with few spikes (Fig. 1A). The mean lengths for carriers and non-carriers were precisely identical at 13.2 ± 0.0 (SE) amino acids. This was a little shorter than averaged from the database^16^. The difference might be explained by the nature of the younger population since CDR-H3 lengths tend to be longer in the elderly^14^. The mean length was found to be longer in adults recruited for the second part study (see below). The top two longest CDR-H3 clones contained 56 and 55 amino acids, respectively. They were identified from the 14-year-old non-carrier in the 2^nd^ family, who also was the oldest child (Table 1). The 5-year-old non-carrier in the 4^th^ family also had two CDR-H3 clones with 54 amino acids.

Compositions of amino acids among all 16 IgG CDR-H3 immune repertoires exhibited very high similarities (Fig. 1B). The most frequently used 6 amino acids were Tyr, Asp, Gly, Ala, Arg, and Ser for all repertoires. These are exactly the same as reported previously^16^. We further assayed mutual resemblances of the repertoire pairs using the Morisita index^17^. This places greater emphasis on more abundant clones (Supplementary Table 2). The results revealed very high variations independent of sibling relationships. There were few common clones with indices up to 0.997~0.999. Differences between pre- and post-vaccination samples from the same individual were lower. However there were still significant dissimilarities among either the carrier (0.955 ± 0.004, SD) or the non-carrier groups (0.945 ± 0.038, SD). In summary, IgG CDR-H3 immune repertoires were distinct in clonal contents, but were similar in length and amino acid compositions regardless of the HBV status.

### Carriers resembled each other in the principal component analysis

**Figure 2:**
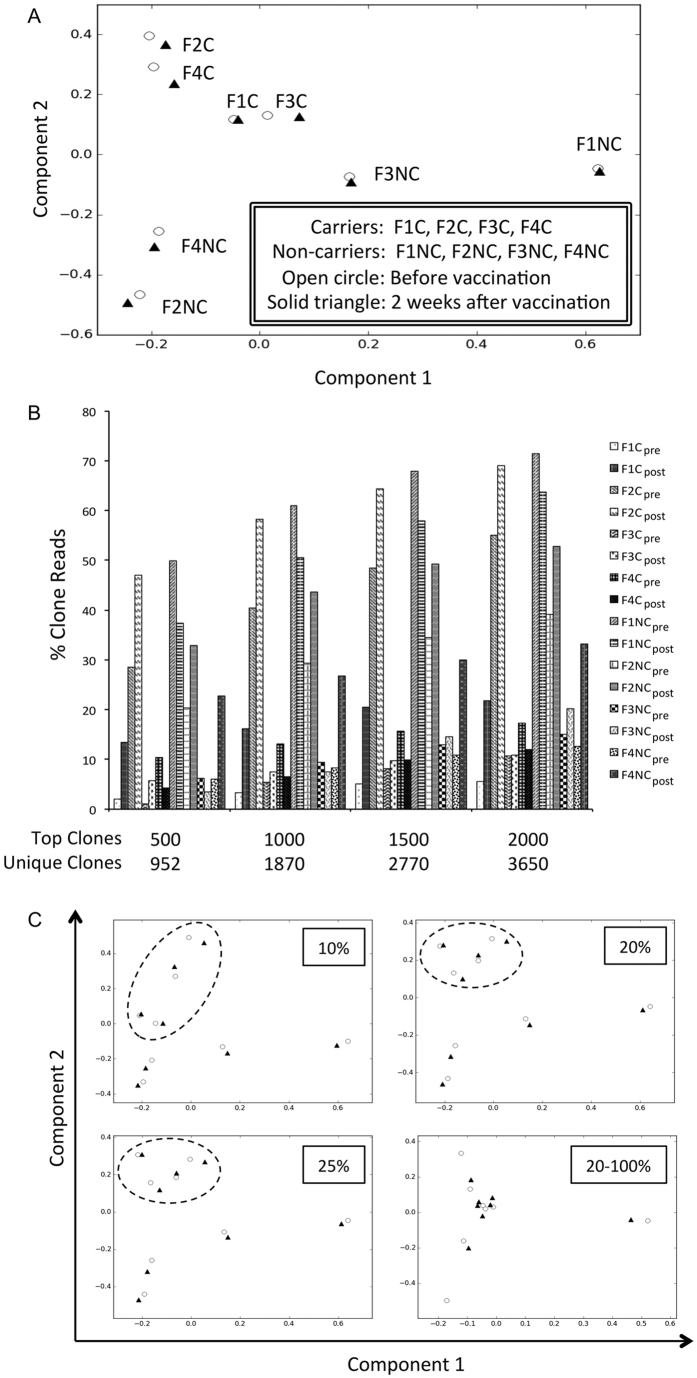
Principal component analysis of IgG immune repertoires. (**A**) Samples from carriers were grouped together. Non-carriers were shown separately. Open circles denote repertoires before vaccination. Solid triangles denote those after vaccination. Repertoires from the same individual were always close to each other, but repertoires between siblings were distant. (**B**) Clones that contributed most to the principal component analysis were also the most abundant. Among the top 500 clones from both components over half of the 16 samples consisted of more than 10% of reads for these sequences. The most significant 3,650 unique CDR-H3s, which comprised merely 0.15% of all clones, trended to accumulate more than one-tenth of reads for every repertoire except one. Over half of the reads of five samples were accountable by these supporting sequences. (**C**) The most abundant 10% of clones were suboptimal in grouping the carrier samples (dotted oval). However the most abundant 20% or 25% of the clones could clearly distinguish the carriers. The trailing 20–100% of clones failed to differentiate the repertoires of carriers from non-carriers.

IgG immune repertoires were represented as Hellinger-transformed^18^ vectors comprising normalized reads of 2,427,148 unique CDR-H3 clones. Principal component analysis (PCA)^19^ effectively reduced millions of dimensions to two components. The discernible implications are that carriers appear to be grouped together but non-carriers are distant from each other (Fig. 2A). Notably, siblings were not closer to each other than unrelated children and vaccination induced little perturbation to PCA projections. Samples from the same individual were always closely aligned. Although the first two components of PCA explained only 7.9% and 7.7% variations in the dataset, this was sufficient to reveal hidden relationships among children with chronic hepatitis B infections, as evidenced by positive values of component 2 for carriers in all.

### Abundant clones discriminated carriers from non-carriers

The top CDR-H3 clones with the highest absolute loading values in the two components of PCA were further evaluated. Reads belonging to these clones occupied a significant portion in the dataset (Fig. 2B). For example, among 952 unique clones pooled from the leading 500 clones in the two PCA components, 9 out of 16 samples already contributed more than 10% of reads to this set of clones. As unique clones were increased to 3,650, they corresponded to only 0.15% of all clones. However more than one-tenth of the reads in all repertoires, except one, could be ascribed to this clone pool. One carrier and two non-carriers had repertoires over half of which had reads that belonged to these abundant clones. It is likely that many of the rare clones could be discarded without compromising the discriminating capabilities of PCA.

Component analyses were accordingly repeated with different percentages of abundant clones (Fig. 2C). The top 10% were suboptimal to distinguish carriers from non-carriers. The resolutions were improved with higher percentages at either 20% or 25%. The trailing 80% of rare clones failed to distinguish repertoires from children with HBV infections. Accordingly, the role of the most abundant 20% of the clones requires further study.

### Biased clones prevailed in carriers

The top 20% of the clones consisted of 481,543 unique sequences. Most were only detected in one child’s pooled repertoire combined from both pre- and post-vaccination samples (Table 2). Among the clones that appeared in two children’s repertoires, fewer than expected were shared by carriers and non-carriers (p < 0.001 in binomial test). A similar observation was also noted for clones with 3 or 4 incidences (p < 0.001 in binomial test). These phenomena could be due to analogous humoral immune reactions only in carriers such that reactive clones make exclusive appearance from non-carriers. Because CDR-H3 sequences with indel-free Hamming distance 1 could be related biologically^9^,^12^,^20^,^21^, a heuristic to efficiently examine the hypothesis of humoral analogy was to cluster CDR-H3 clones with the above Hamming distance criterion. An exhaustive search for connections among the 2-incidence 8,502 clones in carriers and 11,158 clones in non-carriers identified 7,129 and 9,560 independent clusters, respectively.

**Figure 3:**
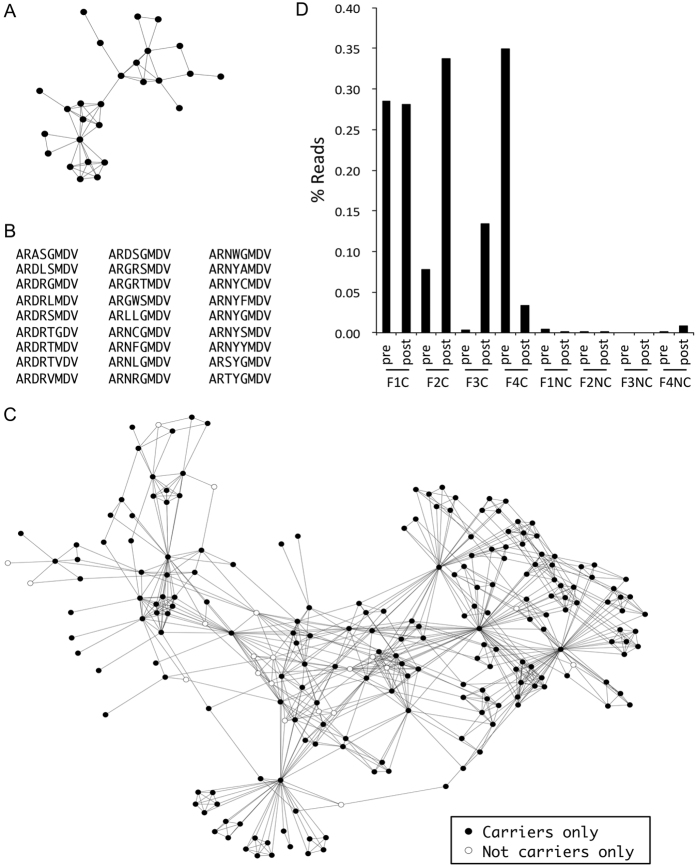
CDR-H3 cluster among carrier children. (**A,B**) A single 27-member cluster was identified from carrier 2-occurrence clones. This cluster was best associated with HBV-infection. Vertices in the graph represent CDR-H3 clones, and connecting edges equal in indel-free Hamming distance 1. (**C**) The 27-member cluster was extended by indel-free Hamming distance 1 to connect other similar sequences among the most abundant 20% of clones. This resolved into a new 222-member cluster. Most members were restricted to carriers only, as depicted in solid circles. (**D**) The extended cluster appeared mostly in the carrier children. Each child had at least one repertoire that contributed more than 0.1% of reads to the connected web.

Under the hypothesis that clusters with more members could be more biologically relevant, the top 0.5% clusters in ranks of member counts from either group were included for feature selections with both linear support vector classification (SVC) and logistic regression (LR) regularized with “l1” penalties in the scikit-learn package^22^ to best categorize infection statuses. The penalty parameters were chosen by minimizing the error rates of 8-fold cross validations to 0.0625 (Supplementary Fig. 2). Out of the 35 clusters in carriers and the 47 clusters in non-carriers, the top 27-member cluster in the infected group was highly supported by both SVC and LR models (Fig. 3A,B). Only 4 clusters in the carriers and 1 cluster in the non-carriers were otherwise weakly supported.

In view of the long duration of chronic infection and iterative nature of B cell immune reactions, we expected that there would be more carrier-associated clones. Based upon the distance heuristic describe above, an additional 195 out of the remaining 481,516 abundant sequences were found within the reach of one Hamming distance. As expected nearly all additional clones belonged to the carrier group rather than the non-carrier group (Fig. 3C). These related sequences contributed 0.1–0.3% reads to the IgG immune repertoires of all carriers in at least one sample (Fig. 3D). The near absence of the huge clone web among non-carriers implied that these connected CDR-H3 sequences provided a remarkable signature of chronic infection in hepatitis B carrier children.

### Vaccination differentially affected diversity of immune repertoires

**Figure 4:**
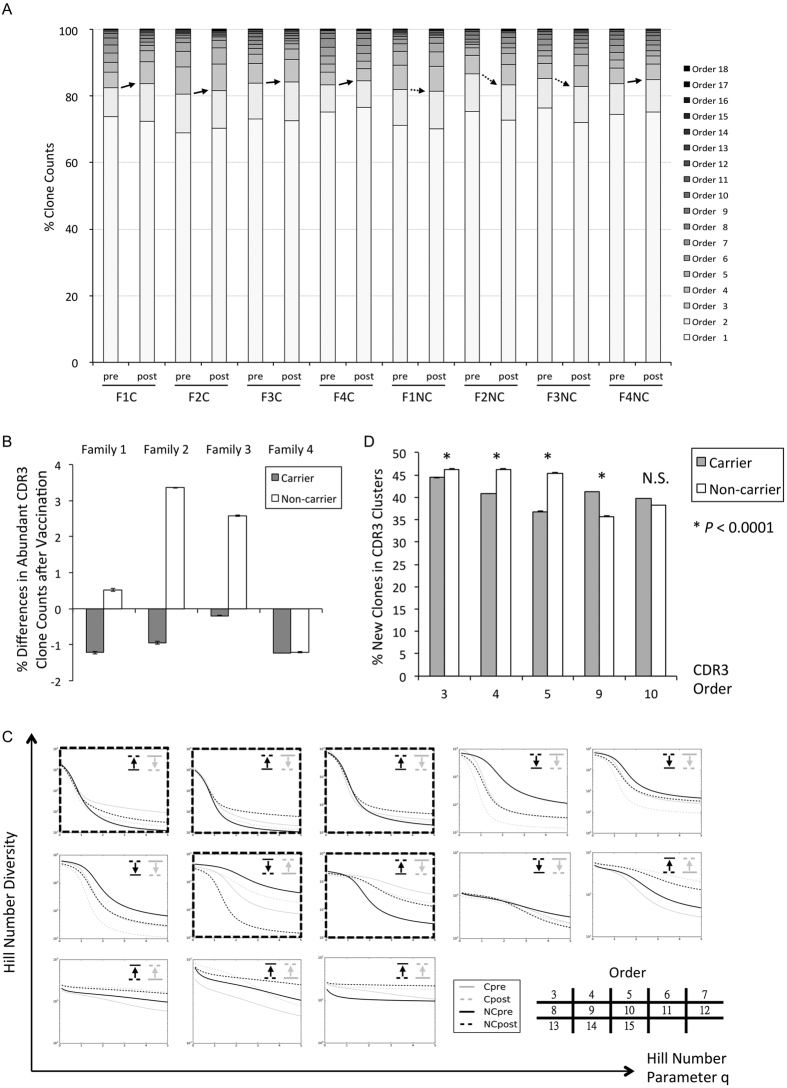
Changes in clonal diversity with vaccination. (**A**) Clone counts in order 3 or higher decreased after vaccination for all the carriers. In contrast, they tended to increase among the non-carriers. The results are shown as the average of 10 rarefactions. (**B**) There was about a 1% decrease of clone counts in order 3 or higher for carriers. This was universal for all rarefaction datasets. This tendency was instead increased for most non-carriers. Error bars show the standard errors, Student’s t-test p < 0.05. (**C**) Diversities of CDR-H3 clusters connected with indel-free Hamming distance 1 in each order were profiled in Hill numbers. Vaccination-induced trends with parameter 2 or above distinguished carriers from non-carriers within order 3, 4, 5, 9, and 10. In order 9 carriers exhibited a positive shift after vaccination. However non-carriers had increased diversities in order 3, 4, 5, and 10. A typical result is shown from one rarefaction dataset. (**D**) Percentages of new clones in post-vaccination CDR-H3 clusters belonging to the five mid-abundance orders were calculated. Non-carriers had a significantly higher percentage in order 3, 4, and 5. Carriers had more new clones in order 9 (p < 0.0001 in Student’s t-Test for all rarefactions). The difference in order 10 was insignificant. The greatest contrast was noted for order 5.

Different impacts to the diversities of CDR-H3 clones were expected with vaccination in non-carriers versus carriers because of immune tolerance^5^. To establish a fair base for comparisons of clone diversities between IgG immune repertoires, random rarefactions were applied to the dataset to normalize sequence reads for all repertoires. Clones were stratified by the base 2 logarithm of reads into 18 orders such that clones with similar functional importance would be grouped together^15^. Clone counts in each order were averaged after 10 rounds of rarefactions (Fig. 4).

We found that orders higher than 2 contained the most abundant 20% of the clones. These exhibited different effects following vaccination (Fig. 4A,B). The post-vaccination repertoires of all the children with chronic HBV infections showed a slight decrease in counts of clones ranked in order 3 or higher, but most of the healthy siblings exhibited an increase (−0.90 ± 0.24 vs. 1.32 ± 1.03 % SE, p < 0.05 in Student’s t-test, Fig. 4B).

Diversities of clones in each order were further characterized in two steps: clusters were constructed first by connecting sequences with indel-free Hamming distance 1 and were subsequently profiled with Hill numbers^23^ for all rarefaction datasets (Supplementary Fig. 3). Post-vaccination effects were categorized according to the curve shifts of Hill numbers with parameter 2 and above such that the most frequent clusters had greater diversity measurements (Supplementary Table 3). Among order 3–10 which constituted the majority of the top one-fifth of clones non-carriers had order 3, 4, 5, and 10 with positive shifts but carriers had only order 9 with such a positive trend (Fig. 4C). Because both carriers and non-carriers reacted similarly in order 11–15 and few clusters existed in order 16–18, vaccination-associated clusters would be less likely present among these orders. Instead differential influences by vaccination were mainly suspected in the five mid-abundance orders exhibiting overall tolerant behaviors with less diversity expansions in carriers’ immune repertoires.

Increases in diversity measurements could result from amplification of pre-existing clones before vaccination or alternatively from contributions of new clones freshly appearing after vaccination. In order to differentiate these two possibilities, the percentages of new clones in post-vaccination CDR-H3 clusters belonging to the five mid-abundance orders were determined (Fig. 4D). Clearly the positive momenta were driven by the presence of new clones such that non-carriers had higher percentages in order 3, 4, and 5 but lower percentages in order 9 (p < 0.00005 in Student’s t-Test for all rarefactions). The most conspicuous difference was located in order 5, where a nearly 10% margin was led by non-carrier children. Inequality in order 10 did not reach statistical significance in any of the rarefaction datasets.

### Vaccination-responding clusters manifested in order 5

The contradictory effects on CDR-H3 diversities as demonstrated in order 3, 4, 5, 9, and 10 among IgG immune repertoires could increase the odds to discover sequences that correlated with specific vaccination responses. Therefore the top 0.5% clusters in ranks of clone counts were tested with both linear SVC and LR models regularized with “l1” penalties to best recognize post-inoculation repertoires in non-carriers. The penalty parameters were chosen by minimizing the error rates after 8-fold cross validations. We found that SVC and LR models for order 3, 4, 9, and 10 disagreed considerably with each other for clusters that best distinguished the post-vaccination repertoires in non-carriers. On the other hand for order 5 the LR models consistently demonstrated the top-ranked 4 clusters by SVC models, but failed to support any of the other candidates (Supplementary Table 4). The significance of these observations was tested with random label permutations for 10,000 runs in each rarefaction. We found that the chance of 4 or more clusters with simultaneous strong support by both models was less than 0.02. The candidate clusters always belonged to those with abundant member clones. None of the clusters that exceeded 0.3% in ranks received nonzero coefficients in either SVC or LR selections (Supplementary Table 4).

**Figure 5:**
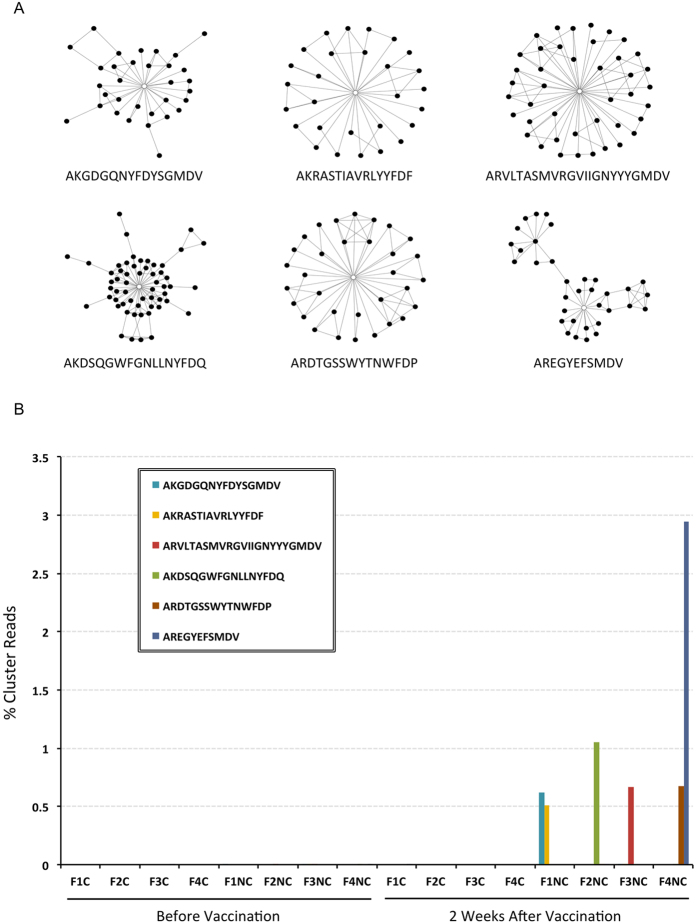
CDR-H3 clusters associated with vaccination in non-carrier children. (**A**) Six CDR-H3 clusters in order 5 were selected by both linear support vector classification and logistic regression. An open circle marks the clone with the highest PageRank score. The corresponding sequence, listed below, was connected to other similar clones with an indel-free Hamming distance of 1. (**B**) The six clusters were further extended with two rounds of Hamming distance 1 as described in the text. Percentages of the reads belonging to the new clusters rose significantly in post-vaccination samples only in the non-carriers. In family 1 and 4 there were two amplified clusters. There was only one new cluster in family 2 and 3. Vaccination was associated with at least a 0.5% increase in cluster reads for all non-carrier children.

Detailed analyses of the dominant candidates in SVC models demonstrated essentially 6 independent clusters out of 10 rarefaction datasets (Supplementary Table 5). Five of the 6 connected graphs presented as bursting stars with the highest PageRank^24^ vertex located at the center mimicking iterative activities from a prototype clone (Fig. 5A). The clusters constructed from rarefactions were extended by incorporating clones within indel-free Hamming distance 1 reach from the original dataset. To further test the specificity of these constructed clusters, a second outreach to include even another one indel-free Hamming-distanced clones was performed. This exhaustive search increased clone counts of clusters to 479, 346, 436, 705, 294, and 849, respectively (Supplementary Fig. S4A). Percentage reads of the six extended clusters correspondingly rose to 0.5% to 3% in post-vaccination repertoires among non-carriers only. Their pre-vaccination samples had fairly low levels of these clones instead (Fig. 5B). Two non-carriers responded polyclonally; there were two clusters at comparable levels. This observation demonstrated the stochastic nature of rarefactions in the analytic framework.

### Analysis of clusters among adults following a booster vaccination

Identical vaccination-responding clusters could be discovered by SVC and LR feature selections without contributions from carriers’ clones in order 5 (data not shown). This made it reasonable to use the analyses scheme for other scenarios as well. To test this possibility, we established a new dataset by collecting samples from 4 healthy adults receiving HBV vaccine boosters (Supplementary Fig. S1A). Each adult had 3 repertoire libraries from 3 time points at prior to, 1-week and 2-weeks after vaccination (Table 1). We found that adults 2, 3, and 4 already had baseline anti-HBs antibodies above the detection threshold. Adult 1 achieved a positive serologic response only after receiving the booster. The libraries were processed through the same pipeline (Supplementary Fig. S1C). The mean length of CDR-H3 was 14.7 ± 0.1 (SE) amino acids. This was longer than in children, as expected (see above).

**Figure 6:**
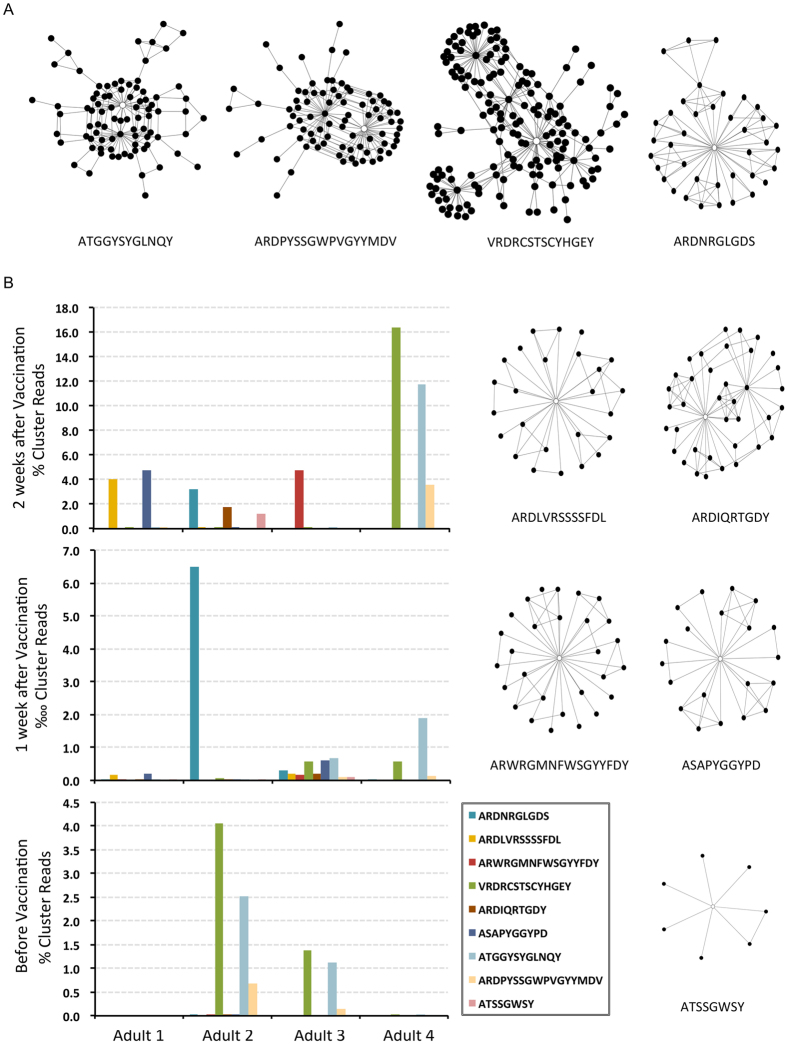
CDR-H3 clusters associated with vaccine boosters among healthy adults. (**A**) Nine CDR-H3 clusters in order 5 were selected by both linear support vector classification and logistic regression. The clone in the open circle had the highest PageRank score. The corresponding sequence is listed below. All clones were connected to similar clones with an indel-free Hamming distance of 1. (**B**) A two-step extension by Hamming distance 1 for the nine clusters was performed as described in the text. Read percentages of the new clusters rose significantly at 2 weeks following vaccination and were detectable for the indicated individuals at 1 week following the booster. All except adult 3 reacted with more than one clone network. None of the adults shared clusters even though the 3-cluster profile from adult 4 was also present at baseline for adults 2 and 3.

Ten rarefaction datasets were prepared for downstream analyses. In the Hill number plots (Supplementary Fig. 5), the baseline counts of unique clones were much higher than those from post-vaccination samples. The observations were illustrated as Hill numbers with parameters approximating zero (see methods). For order 5 most of the 2-week samples exhibited an upward trend as compared to baseline. The same strategy with SVC and LR feature selections depending on the baseline and 2-week repertoires was applied to order 5 clones (Supplementary Table 6), not including the 1-week data. Nine clusters with member counts of 39, 26, 29, 163, 38, 21, 86, 85, and 8 were identified (Fig. 6A and Supplementary Table 7). A similar 2-step indel-free Hamming distance 1 extension as performed for the 6 clusters in Fig. 5B was done to exhaustedly incorporate related clones from the complete adult dataset (Supplementary Fig. S4B). This increased the member counts of the 9 clusters to 529, 327, 523, 2577, 345, 286, 1474, 1144, and 166, respectively. We found that most adults responded polyclonally at 2 weeks after vaccine boosters. This enriched the read percentages up to 16% in the repertoires (Fig. 6B). The 3-cluster profile for adult 4 at week 2 was the same as that for adult 2 and 3 at baseline. Adults 2 and 3 responded to vaccination with new clusters (Fig. 6B). Although clusters were selected independent of 1-week data, corresponding reads were already increased at that time point for indicated adults (Fig. 6B). The gradual enrichment of these clusters implied specific roles they played in response to vaccination. The significance was further addressed using the Spearman rank test for each cluster in terms of read percentages. This collectively reached a p-value of 0.03 with Bonferroni corrections for multiple comparisons.

## Discussion

Effective methods to extract relevant sequences out of CDR-H3 repertoires in response to vaccination would be of great clinical value. Many investigators, in recent years, have addressed this challenge by use of high-throughput sequencing. This is not a simple task because there can be 10^11^ or higher possible receptor sequences among B cells^25^. One strategy to increase the sensitivity and specificity is to pool related clones into clusters that are functionally related^9^,^12^,^20^,^21^. It is now known that sequence similarities among plasmablasts at one week after vaccination are very limited, even between identical twins^1^. The most notable sequence convergence occurs at 2–3 weeks after vaccination from memory B cells^10^. The number of corresponding memory cells can rise up to a hundred fold above baseline at these time points^10^. This would justify a simple heuristic to use abundance sums of related clones at 2 weeks after vaccination to discover the optimum CDR-H3 signals (Supplementary Fig. S1A).

In the current study we identified 6 clusters in children following HBV vaccination (Fig. 5A). None of the clusters were shared among individuals, but two of children appeared to react simultaneously with two clusters (Fig. 5B). Similarly 3 out of 4 adults responded with more than one cluster at 2 weeks after vaccination (Fig. 6B), but none shared the same cluster. Prior investigators have found limited similarities of CDR-H3 sequences after vaccination. For example, specific sequences against tetanus toxoids can be categorized into at least 9 groups^10^; responding sequences after repeated HBV vaccination could be different as well^26^. Because of our small sample size we did not expect to find convergence of reacting clusters. However the 3-cluster profile from adult 4 was also present at baseline for adult 2 and 3 (Fig. 6B). Both of these subjects were known to have significant amounts of antibodies against HBsAg before receiving the boosters. We suspect that these clusters could belong to the same group of protective antibodies. Further functional verification would be required.

One large set of connected CDR-H3 clones was identified in HBV carriers (Fig. 3C). The clone network belonged to the most abundant subset of IgG repertoires, implying that the continuous presence of HBV might cause a persistent expansion of related CDR-H3 sequences. It is known whether antibodies against HBcAg are a hallmark of prior HBV exposure^3^. Therefore the relationship of this clone cluster with anti-HBc antibodies is worthy of further investigation. In this study we did not attempt to associate CDR-H3 sequences with antibodies against HBeAg. In view of the essential role of such antibodies to produce a stage shift for hepatitis B carriers (e conversion)^3^, it would be of interest to compare IgG repertoires both before and after e conversions.

Hill numbers^23^ have been widely used to evaluate ecological diversities. In the current study we adapted the formula by comparing CDR-H3 clusters to ecological species. Differences were detected in Hill numbers between carrier and non-carrier children in relation to vaccination responses (Fig. 4C). Greiff *et al*. independently found that Hill numbers were applicable to assay immune repertoires under various clinical conditions^27^. Our findings were based on clusters among sorted orders of individual clones, allowing more focused linkage to clinical status. When parameters are set to zero, Hill numbers provide counts of cluster numbers. We found that the baseline counts in adults were always much higher than those in post-vaccination samples (Supplementary Fig. 5). However this was not the case for children (Supplementary Fig. 3). The difference between children and adults might be explained by a more diversified clone profile in an age-related IgG repertoire baseline and a more active expansion of memory B cells in response to vaccination. This notion is supported by both the higher read percentages of reacting clusters and a much more complex plots of clone networks in Fig. 6.

## Methods

### Ethics, consent and permissions

All experimental protocols were approved by both the Institutional Review Board of National Health Research Institutes and the Institutional Review Board of En Chu Kong Hospital. Study methods were carried out in accordance with the relevant guidelines. Informed consents were obtained from all subjects.

### Case enrollment and blood sampling

For the first part of study in children, HBeAg positive carrier and HBsAg negative non-carrier siblings born to an HBeAg positive mother were enrolled from 4 families (Table 1). None of the children had active hepatitis. Each received a 1 ml intramuscular injection of ENERGIX-B (GlaxoSmithKline, UK). Two blood samples of 2.5 ml each were collected into PAXgene Blood RNA tubes (PreAnalytiX, CH) before and 2 weeks after vaccination (Supplementary Fig. S1A), respectively. Tubes were kept at room temperature for at least 2 hours before RNA extraction or −80 °C storage. In the second part of study 2.5 ml whole blood was collected from four healthy adults at baseline and at 1 and 2-weeks after vaccination (Supplementary Fig. S1A). Specimens were processed in the same manner as for children.

### Software and hardware

Software packages including PEAR^28^, Python^29^, Numpy & SciPy^30^, IPython^31^, scikit-learn^22^, Pandas^32^, Matplotlib^33^, PyOpenCL^34^, and igraph^35^ were deployed on a 2013 Mac Pro equipped with 3.7 GHz Quad-Core Intel Xeon E5, 64 GB memory, and two AMD FirePro D700 6 GB graphics cards.

### Preparation of IgG immune repertoire for next generation sequencing

The experimental procedures were summarized in Supplementary Fig. S1B. Total RNA was extracted with PAXgene Blood miRNA Kit (QIAGEN). 1.2 μg was reverse-transcribed with an IgG constant region specific primer (Supplementary Table 1) with SuperScript III Reverse Transcriptase and RNaseOUT (Life Technologies), going through 65 °C 5 min, on ice at least 1 min, 55 °C 60 min, and final 70 °C 15 min.

Normalized cDNA were multiplex amplified with a set of forward V-primers from known IgG alleles^36^ that were Primer 3^37^ optimized by the same parameters and two reversed primers (Supplementary Table 1). All forward primers shared similar amplification efficiencies with linear correlations to cDNA concentrations across 5 logs and had low backgrounds as well as specific products in pilot optimizations. The reaction condition was 98 °C 2 min, 15 cycles of 98 °C 80 sec + 60 °C 60 sec + 65 °C 30 sec, 10 cycles of 94 °C 30 sec + 65 °C 90 sec, and final 65 °C 5 min with AccuPrime *Taq* DNA Polymerase High Fidelity (Life Technologies).

The product was amplified in a second PCR by the same polymerase with double-indexed P5 and P7 primers^38^ (Supplementary Table 1) under the condition 94 °C 2 min, 10 cycles of 94 °C 30 sec + 60 °C 30 sec + 68 °C 40 sec, 10 cycles of 94 °C 30 sec + 72 °C 90 sec, and final 72 °C 5 min. Products were sieved by 2% agarose gel under 30 V x 8 hours or 60 V ×4 hours and the target bands around 300 bp were eluted with MinElute Gel Extraction Kit (QIAGEN) before 150 bp paired-end sequencing (NextSeq, Illumina, USA). Raw reads were deposited to European Nucleotide Archive under the accession number PRJEB9332 [ENA: PRJEB9332].

### Immune repertoire in amino acids

Demultiplexed raw sequences were processed in Python as outlined in Supplementary Fig. S1C. Briefly, sequences were paired with PEAR^28^; poor quality reads were removed at this step with default parameters, including a Phred filter score at 33. Reads must have no unknown ‘N’ nucleotides as well as matched end sequences on both 5′ and 3′ terminals to the amplifying primers. The expected CDR-H3 region as implicated by matched V-primers had to contain multiplicities of three nucleotides without stop codons. The N-end of translated amino acids from position 100–C104 had to align well to anticipated sequences as suggested by matched V-primers and the C-end had to have the W118 as well as the following GXG signatures, where X denotes any amino acids^8^. At last CDR-H3 without the minimal length of 2 amino acids were discarded. In average 53.8 ± 3.8% and 44.8 ± 4.4% (SD) of raw sequences in children’s and adults’ datasets, respectively, passed the above criteria.

### Morisita index

For repertoire 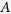 and 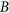 with clone normalized frequencies *F*_*A*_ and 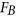, the Morisita dissimilarity index^17^ is a measure to quantify the distance between two sets of clone sequences. This measure is dominated mainly by abundant clones; relatively rare clones have little effect, even if there are many of them. The measure (M) was calculated as


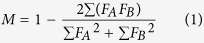


### Principal component analysis with immune repertoires as transformed clone percentages

There were overall 2,427,148 unique CDR-H3 clones from all 16 children’s samples. The normalized clone frequencies for each sample were Hellinger transformed^18^ before principal component analysis^19^ in SciPy. Clones in each repertoire were sorted by read frequencies and the top percentages of abundant clones were identified accordingly.

### Binomial tests for biased clone prevalence

Given 4 carriers and 4 non-carriers, the combinatorial probability for one clone to belong to both groups is calculated with Binomial cumulative tests as


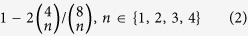


### Rarefaction and cluster identification

Samples with more reads were randomly resampled with Python to match the repertoire with minimal read counts in the indicated dataset (Table 1). Rarefactions were performed 10 times and saved for downstream analyses. Clusters were identified in two steps. Indel-free Hamming distances^39^ between clone pairs were calculated into an adjacency matrix in Python, which was used to initiate a graph in igraph discarding edges weighted higher than distance 1. Cluster-associated functions in igraph were applied to discover independent clusters and to calculate associated PageRank scores^24^.

### Clone orders and cluster diversity in Hill numbers

Without loss of generality, a clone with read count 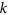 was classified into order 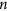 if 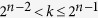. In each order clones with the same HBV status and vaccination history were pooled first. We defined clusters within each clone pool by an adjacency matrix, as described above. We then used Hill numbers, i.e. effective number of clusters in this case, to quantify cluster diversity. Pre- and post-vaccination cluster diversities in Hill numbers (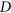)^23^ were calculated following the formula with frequencies normalized in each order:


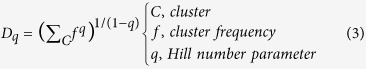


The parameter 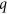 determines each measure’s sensitivity to normalized frequencies. The measure of 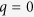 (the total diversity) counts clusters equally without regard to their normalized frequencies. The measure of 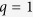 (Shannon diversity, the exponential of Shannon entropy^40^) counts clusters in proportional to their normalized frequencies, and thus can be interpreted as the number of common clusters in the data. The measure of 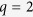 discounts all but the dominant clusters and can be interpreted as the number of dominant clusters in the data. The plot of 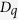 with respect to the parameter 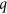 is referred to as a “diversity profile” in ecological science. The profile is generally a decreasing function. The slope of the curve reflects the unevenness of cluster-normalized frequencies. The more uneven the distribution of normalized frequencies, the more steeply the curve declines. Graphs with 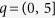 were generated with igraph and Matplotlib (Fig. 4C; Supplementary Figs 3 and 5).

### Cluster selection with support vector classification and logistic regression

Linear support vector classification and logistic regression from Scikit-learn were used to select clusters with “l1” penalties. Hellinger transformed frequencies served as independent variables for classification models. Penalties were chosen by minimizing errors in 8-fold cross validations (Supplementary Fig. 2). In choosing clusters marking chronic HBV infections (Fig. 3), repertoires from carriers regardless of vaccination were labeled positive and penalty parameter was set to 1,000. Carrier and non-carrier clone pools out of the 2-occurrence CDR-H3 sequences (Table 2) were used to define clusters with indicated adjacency matrices. The top 0.5% of clusters from both clone pools as ranked from high to low member counts were included for evaluation. In the case of picking clusters for vaccine responses (Fig. 5), only post-inoculation repertoires from non-carriers were labeled positive and the “l1” penalty parameter was set to 100. The most abundant 0.5% of non-carrier clusters in order 5 were statistically tested, which were identified from pooled clones of non-carriers independent of vaccination history. Significance in the children’s dataset was ascertained by random label permutations for 10,000 runs in each rarefaction, where chances of 4 or more clusters gaining dual strong support with all parameters higher than 20 were calculated. The 9 clusters from the adult dataset were derived using the same strategy.

## Additional Information

**How to cite this article**: Chang, Y.-H. *et al*. Network Signatures of IgG Immune Repertoires in Hepatitis B Associated Chronic Infection and Vaccination Responses. *Sci. Rep*. **6**, 26556; doi: 10.1038/srep26556 (2016).

## Supplementary Material

Supplementary Information

## Figures and Tables

**Table 1:** Basic profiles of subjects and samples.

Symbol	Family	Carrier	Vaccinated	Read #	Clone #	Age	Sex
F1C_pre_	1	Y	N	2,396,273	218,635	13	F
F1C_post_	1	Y	Y	2,343,662	117,953	–	–
F1NC_pre_	1	N	N	2,905,928	159,348	11	M
F1NC_post_	1	N	Y	2,562,822	185,642	–	–
F2NC_pre_	2	N	N	3,025,044	240,809	14	M
F2NC_post_	2	N	Y	2,083,647	165,250	–	–
F2C_pre_	2	Y	N	2,761,308	177,760	12	F
F2C_post_	2	Y	Y	2,308,574	111,368	–	–
F3C_pre_	3	Y	N	2,629,391	138,275	10	M
F3C_post_	3	Y	Y	1,483,239	93,570	–	–
F3NC_pre_	3	N	N	1,409,905	128,753	4	F
F3NC_post_	3	N	Y	1,721,008	99,387	–	–
F4NC_pre_	4	N	N	2,240,377	189,501	5	M
F4NC_post_	4	N	Y	1,702,443	122,475	–	–
F4C_pre_	4	Y	N	1,545,189	159,415	3	F
F4C_post_	4	Y	Y	1,670,657	182,713	–	–
A1_pre_	–	N	N	1,548,623	59,456	42	F
A1_week1_	–	–	1 week	1,655,284	61,541	–	–
A1_week2_	–	–	2 weeks	1,268,812	52,285	–	–
A2_pre_	–	N	N	1,569,305	71,311	35	F
A2_week1_	–	–	1 week	1,108,712	49,262	–	–
A2_week2_	–	–	2 weeks	2,473,612	75,467	–	–
A3_pre_	–	N	N	960,640	34,528	47	F
A3_week1_	–	–	1 week	1,039,772	44,543	–	–
A3_week2_	–	–	2 weeks	1,214,046	35,924	–	–
A4_pre_	–	N	N	952,577	35,765	37	F
A4_week1_	–	–	1 week	1,221,307	56,213	–	–
A4_week2_	–	–	2 weeks	913,435	44,986	–	–

**Table 2:** Distributions of abundant clones among carriers and non-carriers.

Incidences	Total Clone #	Carrier Alone	Non-carrier Alone	Both		p-Value*
				Data	Model	
1 out of 8	458,171	222,661	235,510	0	0	1.00
2 out of 8	22,250	8,502	11,158	2,590	12,715	<0.001
3 out of 8	973	78	241	654	834	<0.001
4 out of 8	119	1	11	107	116	<0.001
5–8 out of 8	30	0	0	30	30	1.00

^*^p-Value was calculated with binomial tests.
