# Supplementary material for: Network Signatures of IgG Immune Repertoires in Hepatitis B Associated Chronic Infection and Vaccination Responses
Source: Sci Rep. 2016 May 25;6:26556. doi: 10.1038/srep26556 (PMC4879636; doi:10.1038/srep26556)
Supplement: Supplementary Information [file srep26556-s1.pdf]

Title

Network Signatures of IgG Immune Repertoires in Hepatitis B Associated Chronic Infection and Vaccination Responses

Authors

Ya-Hui Chang, Hui-Chung Kuan, T. C. Hsieh, K. H. Ma, Chung-Hsiang Yang, Wei-Bin Hsu, Shih-Feng Tsai, Anne Chao, & Hong-Hsing Liu

Supplementary Figure and Table Legends

Supplementary Figure 1. Overview of the study design, construction of the immune repertoires, and the bioinformatic pipeline used to prepare CDR-H3 profiles of the amino acids. (**A**) Two blood samples were obtained from the children and three from the adults enrolled in the study. The first sample was obtained prior to receipt of the HBV vaccine; the second sample was 2 weeks later. For adults an additional sample at 1 week after vaccination was collected. (**B**) 1st-strand cDNA enriched IgG templates with a primer hybridizing specifically to the constant region. The PCR was followed by a set of forward primers targeting various V exons (V-primers) and universal reversed primers. 2nd PCR incorporated double indices at both termini and P5/P7 sequences for the Illumina platform. (**C**) Raw reads in fastq were paired first, and those with ambiguous 'N' nucleotides or those without perfectly matched terminal sequences to PCR primers were discarded. CDR-H3 regions were translatable without stop codons and bridging amino acids from position 100 to C104 had to be well aligned to known sequences. Reads without the characteristic W118 and with CDR-H3 lengths shorter than 2 amino acids were excluded.

Supplementary Figure 2. Error rates in 8-fold or 2-out cross validation with "l1" or "l2" penalties. (**A-B**) Error rates rapidly declined to the lowest 0.0625 with increasing "l1" penalties, and plateaued after penalty parameters passed 1000 for both support vector classification and logistic regression models. (**C-D**) Error rates with "l2" penalties declined slower than those with "l1" penalties. Plateaus were reached when penalty parameters surpassed 10,000 and 100,000

for the support vector classification and the logistic regression models, respectively.

Supplementary Figure 3. Diversity profiles of CDR-H3 clusters in Hill numbers for clone order 3-17 across 10 rarefaction datasets in the children. (**A-J**) CDR-H3 clusters connected with indel-free Hamming distance 1 were constructed in each order from pooled clones with the same infection status and vaccination history. Diversity profiles in Hill numbers were plotted with parameters ranging from 0 to 5. The trends with vaccination were determined by Hill numbers derived from parameter 2 and above such that abundant clusters were favored. In order 3, 4, 5, 9, and 10 cluster diversities shifted distinctly for carriers and non-carriers. Carriers gained a positive turn only in order 9 but non-carriers acquired the upward momenta in all other 4 orders.

Supplementary Figure 4. Graphs of extended clusters associated with vaccination. **(A)** Two-step extension by Hamming distance 1 was applied to rarefaction-derived 6 clusters in children. This enriched the member counts to 479, 346, 436, 705, 294, and 849, respectively. **(B)** 9 clusters related to vaccination response in adults were exhaustedly enriched in two steps with clones within Hamming distance 1 reach to make new clusters containing 529, 327, 523, 2577, 345, 286, 1474, 1144, and 166 members, respectively.

Supplementary Figure 5. Diversity profiles of CDR-H3 clusters in Hill numbers for clone order 3-15 across 10 rarefaction datasets in adults. (**A-J**) In each order pooled CDR-H3 clones from the same timing points after vaccination were used to define clusters with indel-free Hamming distance 1. Diversity profiles in Hill numbers were plotted with parameters ranging from 0 to 5. The baseline Hill numbers with parameter 0 were always much higher than after vaccination. In order 5 the 2-week curves were mostly above the baseline curve for parameter 2 or larger.

Supplementary Table 1. Primer sequences. List of oligonucleotides used in reverse transcription, repertoire preparation, and index incorporation.

Supplementary Table 2. Dissimilarities between samples in measures of the Morisita Index. Values closer to unity in the sample pairs are more dissimilar. Similarities are color coded in the background for better visualization.

Supplementary Table 3. Vaccination-associated shifts of CDR-H3 cluster diversities among carrier and non-carrier children in all rarefaction datasets. Those with increased diversities were labeled with '+', and those with decreased diversities were marked with '-'. The final determination for each order was averaged across all datasets. The carriers and non-carriers were distinctly different in order 3, 4, 5, 9, and 10 (shown in grey).

Supplementary Table 4. Coefficients of both support vector classification (SVC) and logistic regression (LR) models across all rarefaction datasets in children. The top 0.5% of non-carrier clusters with higher member counts were examined. The significant leading 4 clusters in SVC models are shown in dark grey and the corresponding LR coefficients are shown in the same hue. SVC models always supported 4 clusters strongly with occasional minor inclusions of a 5th cluster (light grey). The LR models agreed with SVC models well such that the same 4 clusters gained the highest coefficient values nearly in all rarefactions. Other clusters with non-zero values in LR models are labeled in light grey. In both models there were no clusters beyond the top 0.3% that had coefficients greater than zero.

Supplementary Table 5. Sequences of six CDR-H3 clusters that best signified the post-vaccination samples from non-carrier children. The representative sequences with the highest PageRank scores are listed above. The complete sets are listed at the bottom. In between numbers denoted the ranks from high to low in the SVC models for each rarefaction. In the final dataset the secondly supported cluster did not appear in other datasets and was discarded.

Supplementary Table 6. Coefficients of both support vector classification (SVC) and logistic regression (LR) models across all rarefaction datasets in the adults. The top 0.5% clusters with higher member counts were examined. The significant leading 4 clusters in SVC models are marked in dark grey and the corresponding LR coefficients are shown in the same hue. SVC models always strongly supported 4 clusters with occasional minor inclusions of one or two other clusters (shown in light grey). LR models were in good agreement with SVC models such that the same 4 clusters gained the highest coefficient values in all rarefactions. Other clusters with non-zero values in LR models are labeled in light grey. The modeling used the data from baselines and 2-week samples without inclusions of the data from 1-week samples.

Supplementary Table 7. Sequences of nine CDR-H3 clusters that best signified the post-vaccination samples in adults. The representative sequences with the highest PageRank scores are listed above, and the complete sets are listed at the bottom. The in between numbers denote the ranks from high to low in the SVC models for each rarefaction.

Supplementary Fig. 1

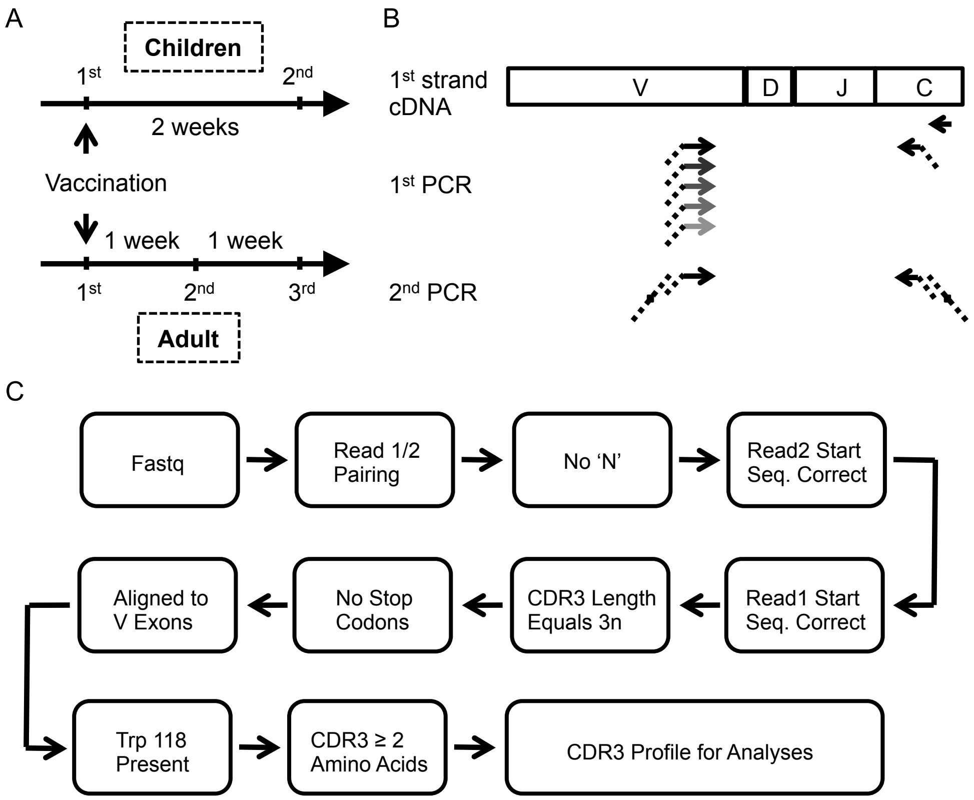

Supplementary Fig. 2

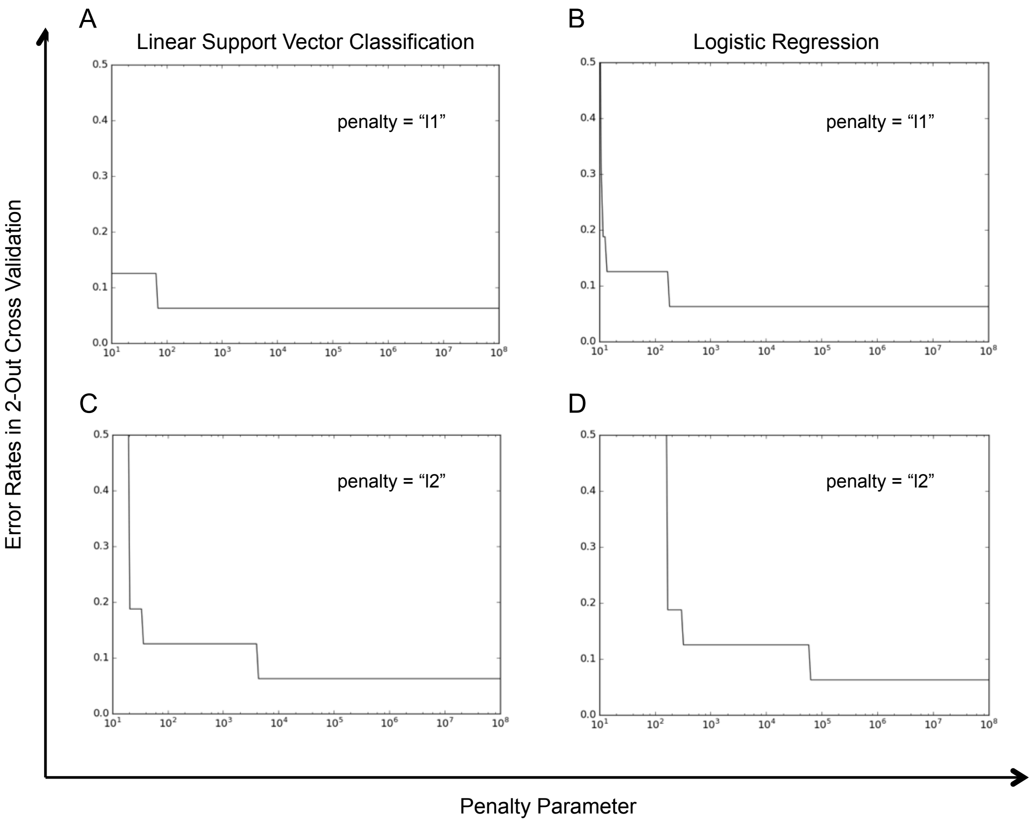

Supplementary Fig. 3

A

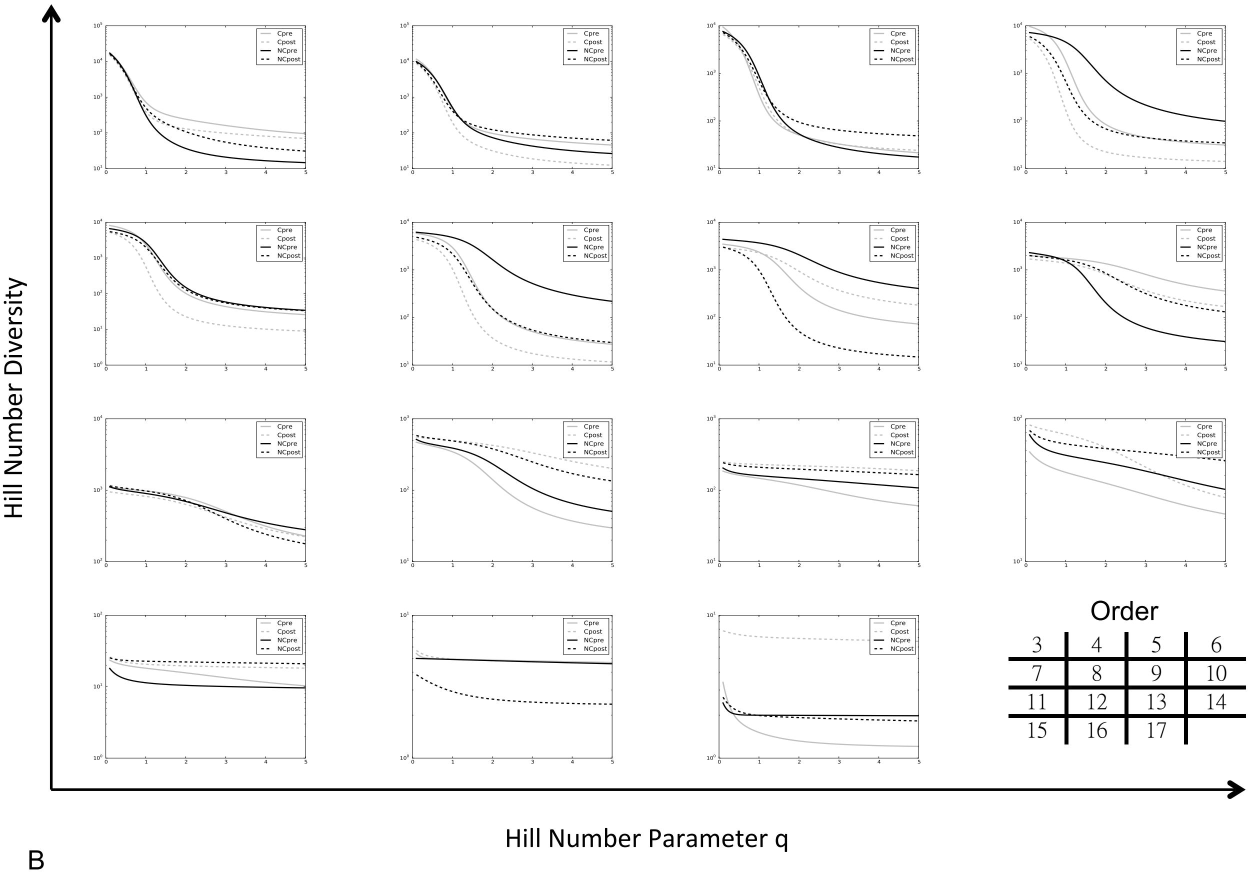

B

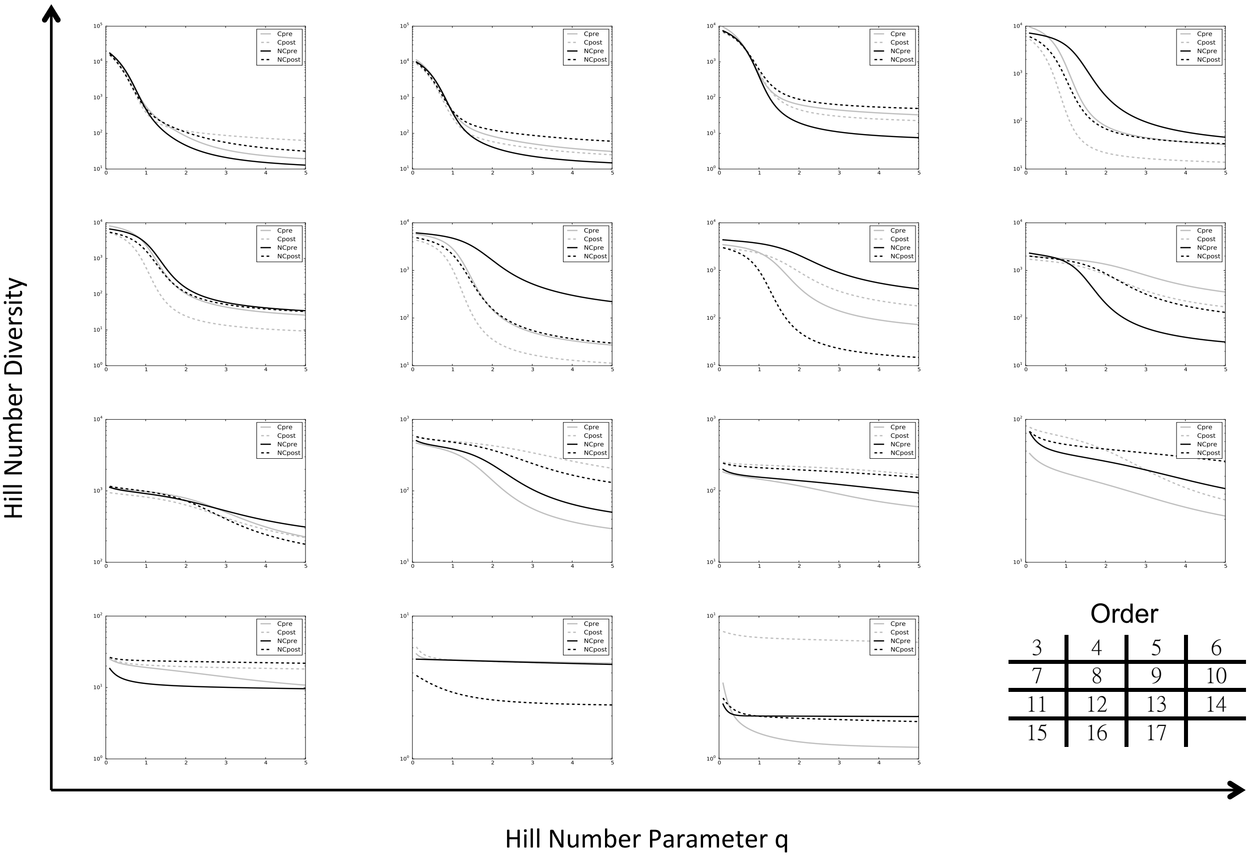

C

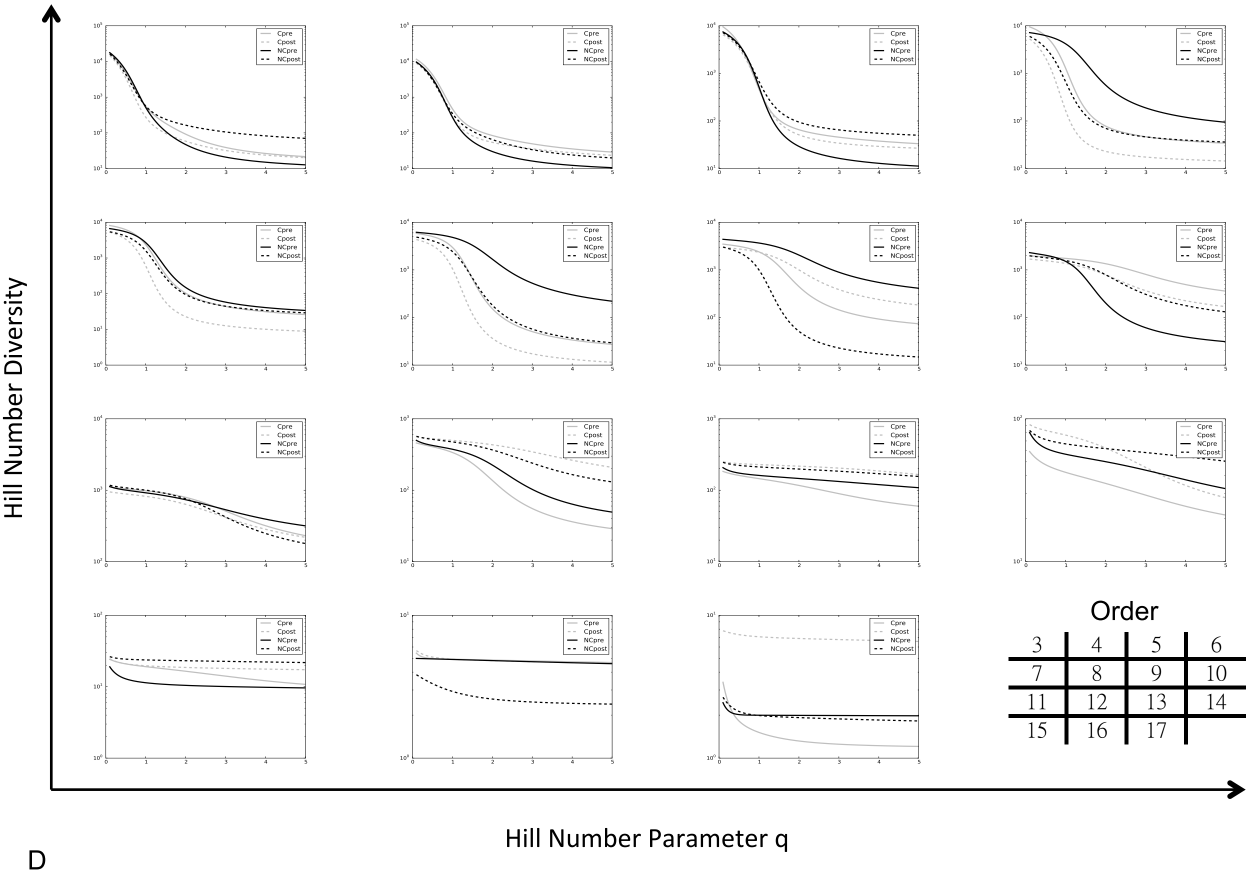
Hill Number Diversity
Cpre
Cpost
NCpre
NCpost
Order
3 4 5 6
7 8 9 10
11 12 13 14
15 16 17
Hill Number Parameter q

D

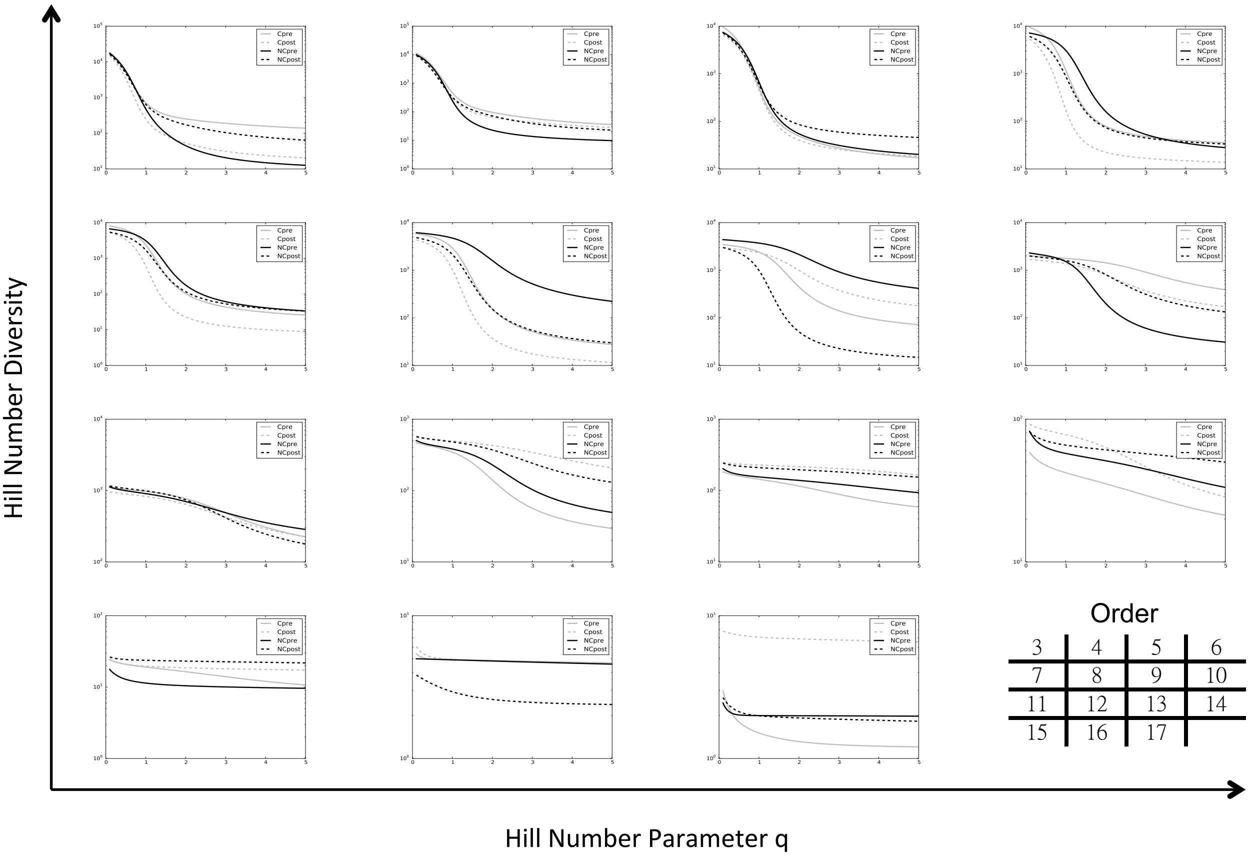
Hill Number Diversity
Cpre
Cpost
NCpre
NCpost
Order
3 4 5 6
7 8 9 10
11 12 13 14
15 16 17
Hill Number Parameter q

E

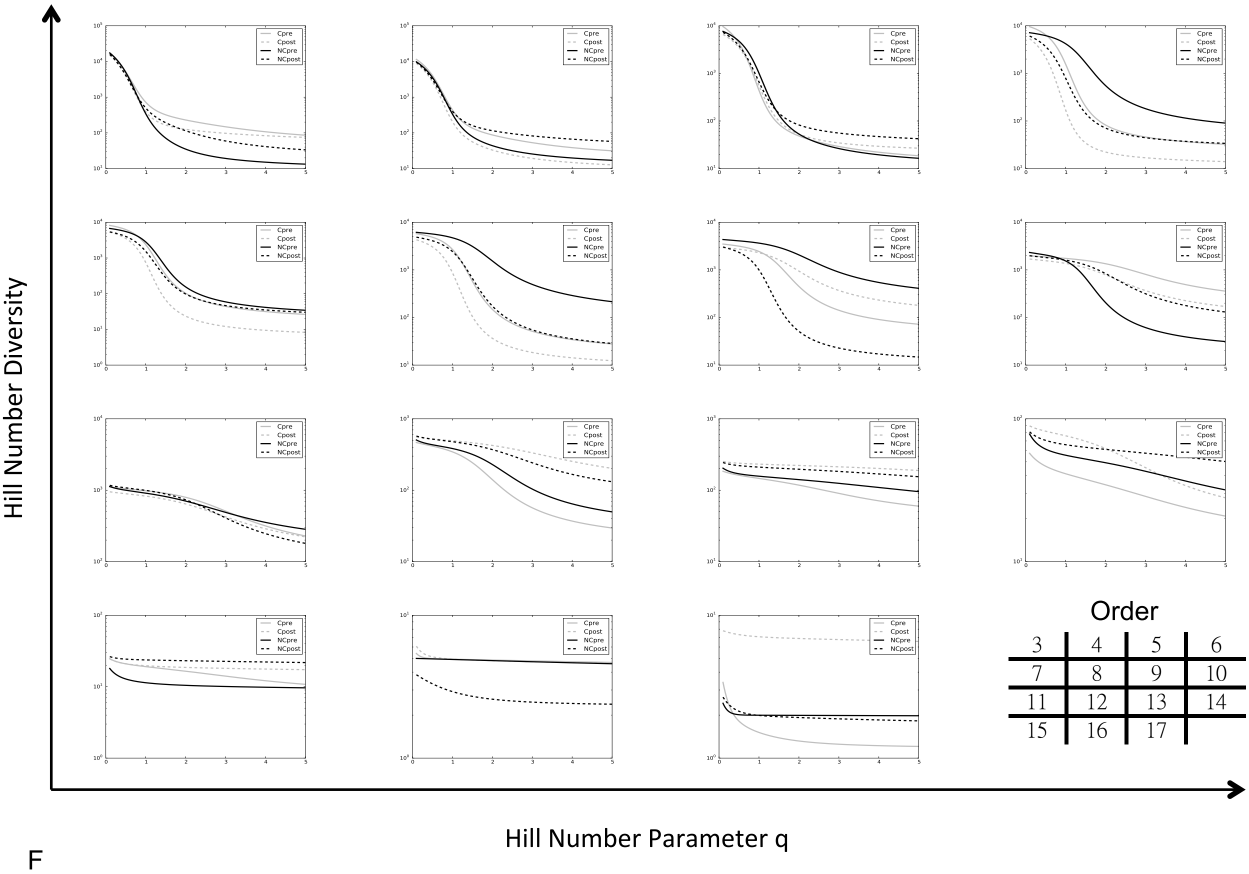
Hill Number Diversity
Cpre
Cpost
NCpre
NCpost
Order
3 4 5 6
7 8 9 10
11 12 13 14
15 16 17
Hill Number Parameter q

F

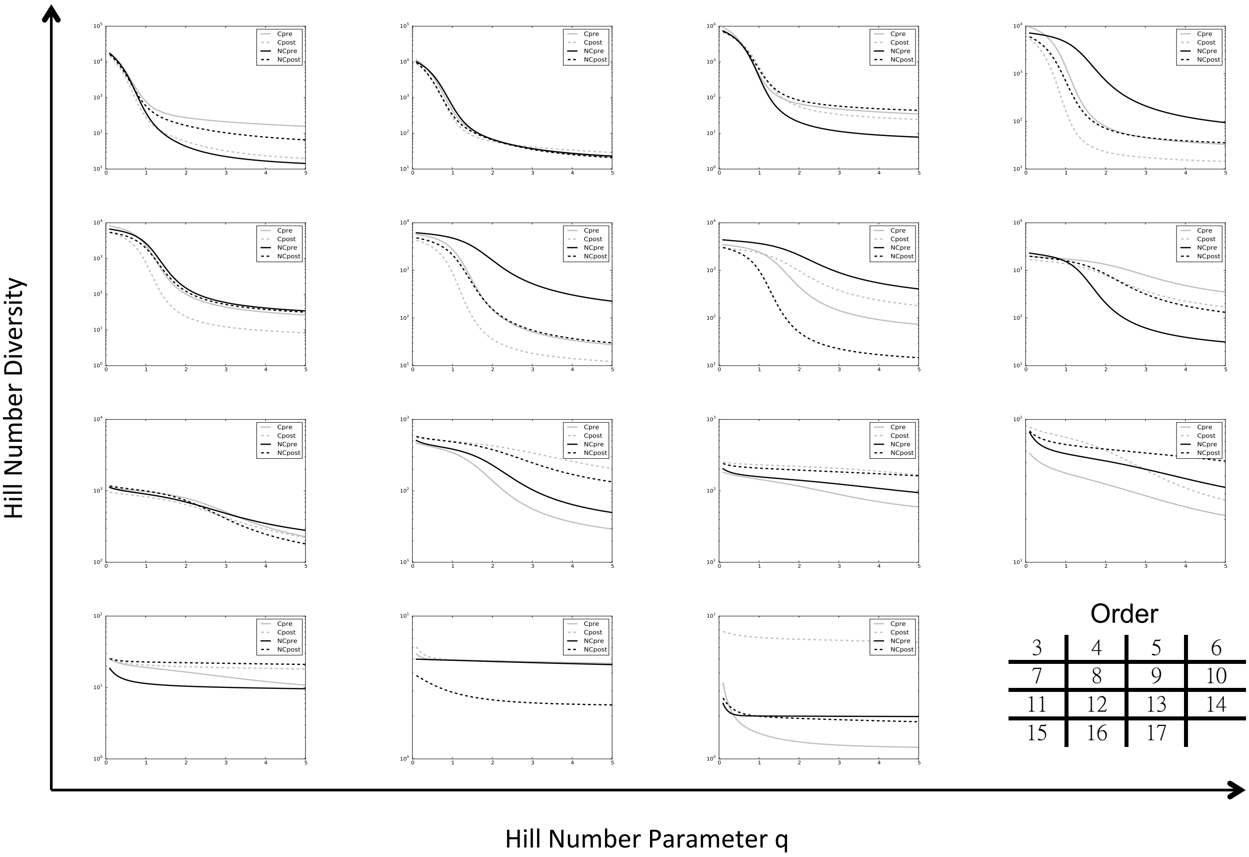
Hill Number Diversity
Cpre
Cpost
NCpre
NCpost
Order
3 4 5 6
7 8 9 10
11 12 13 14
15 16 17
Hill Number Parameter q

G

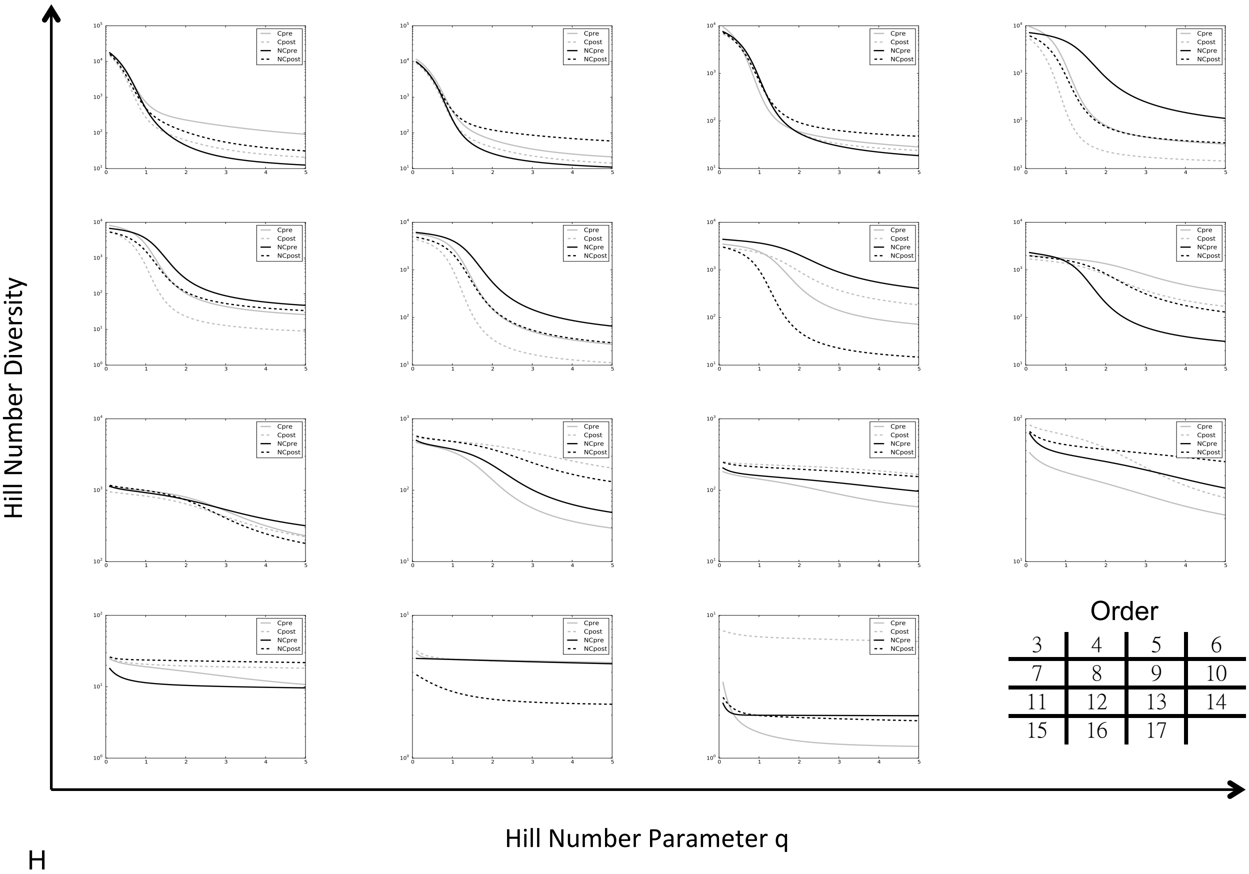
Hill Number Diversity
Hill Number Parameter q
Cpre
Cpost
NCpre
NCpost
Order
3 4 5 6
7 8 9 10
11 12 13 14
15 16 17

H

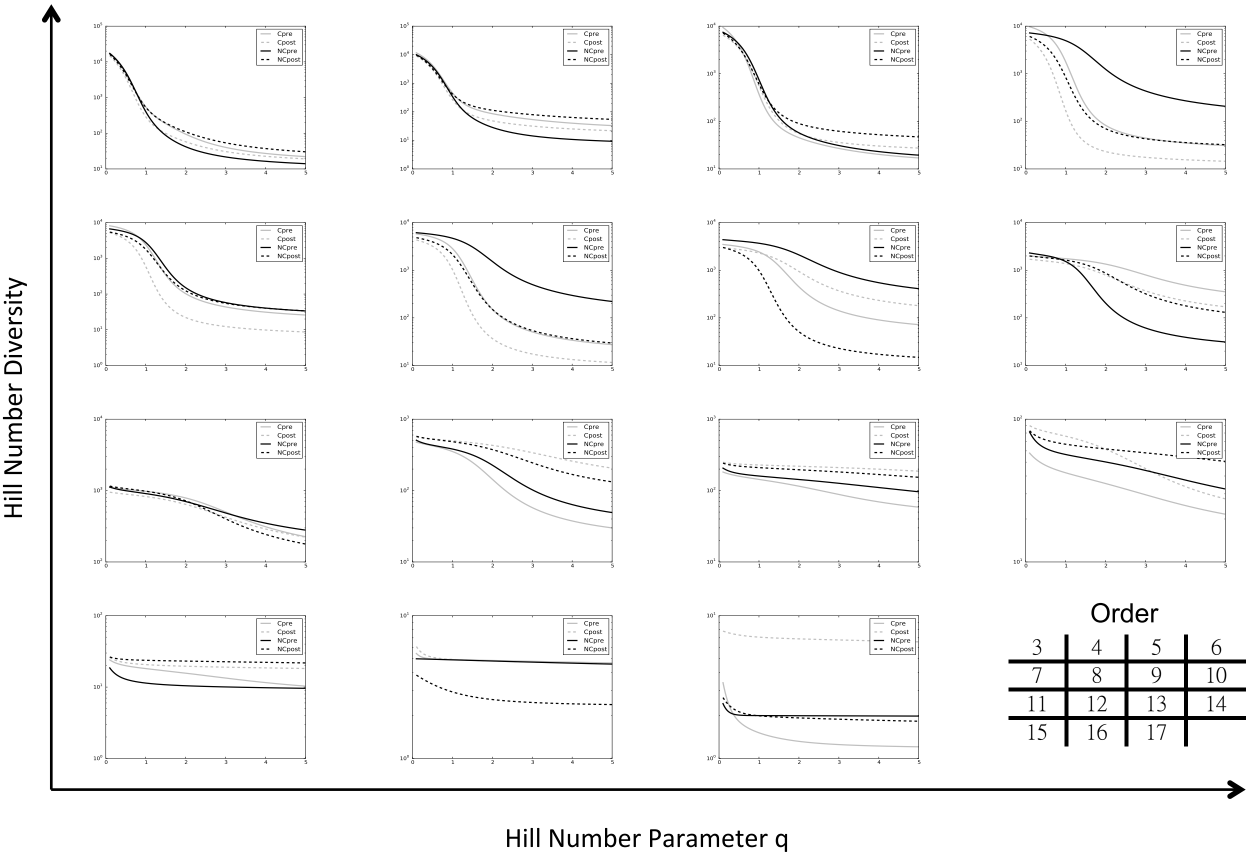
Hill Number Diversity
Hill Number Parameter q
Cpre
Cpost
NCpre
NCpost
Order
3 4 5 6
7 8 9 10
11 12 13 14
15 16 17

I

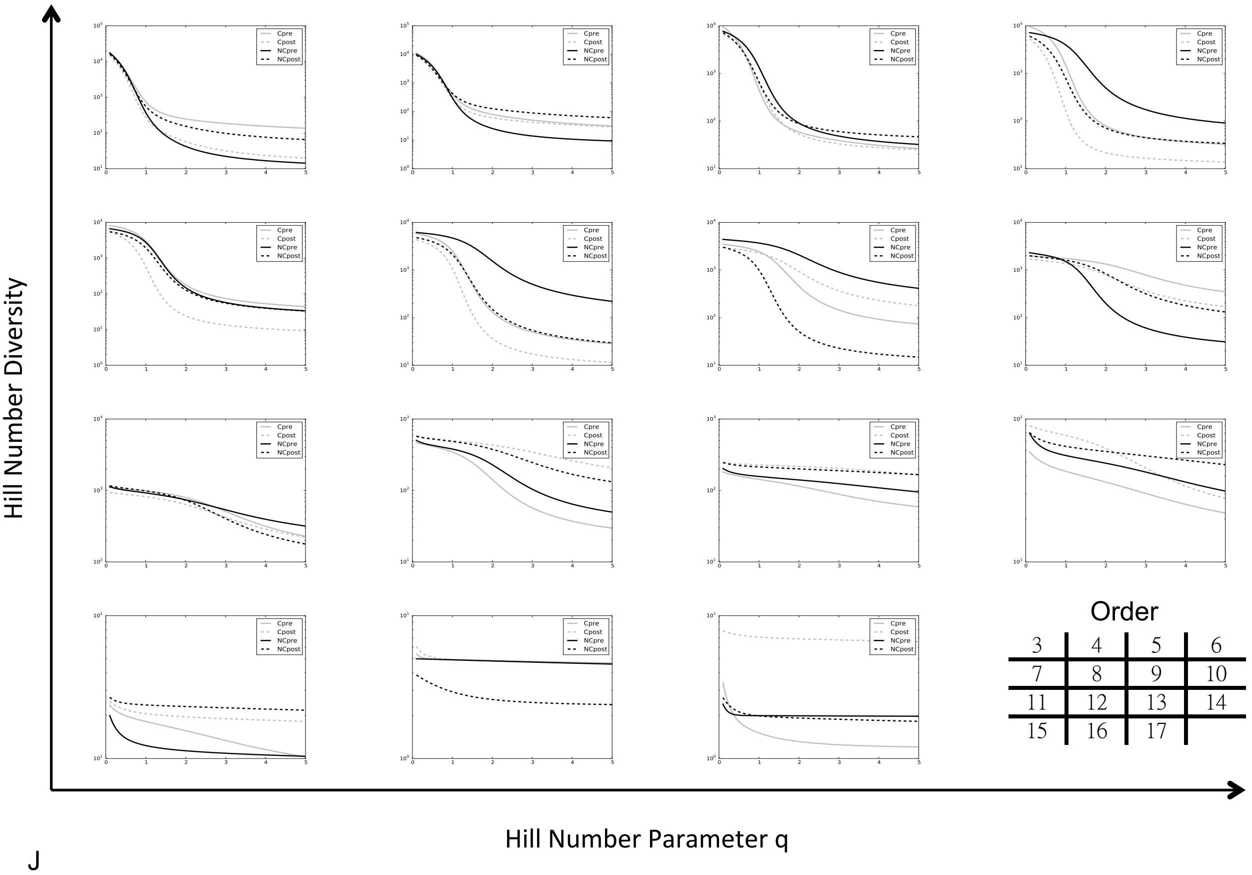
Hill Number Diversity
Hill Number Parameter q
Cpre
Cpost
NCpre
NCpost
Order
3 4 5 6
7 8 9 10
11 12 13 14
15 16 17

J

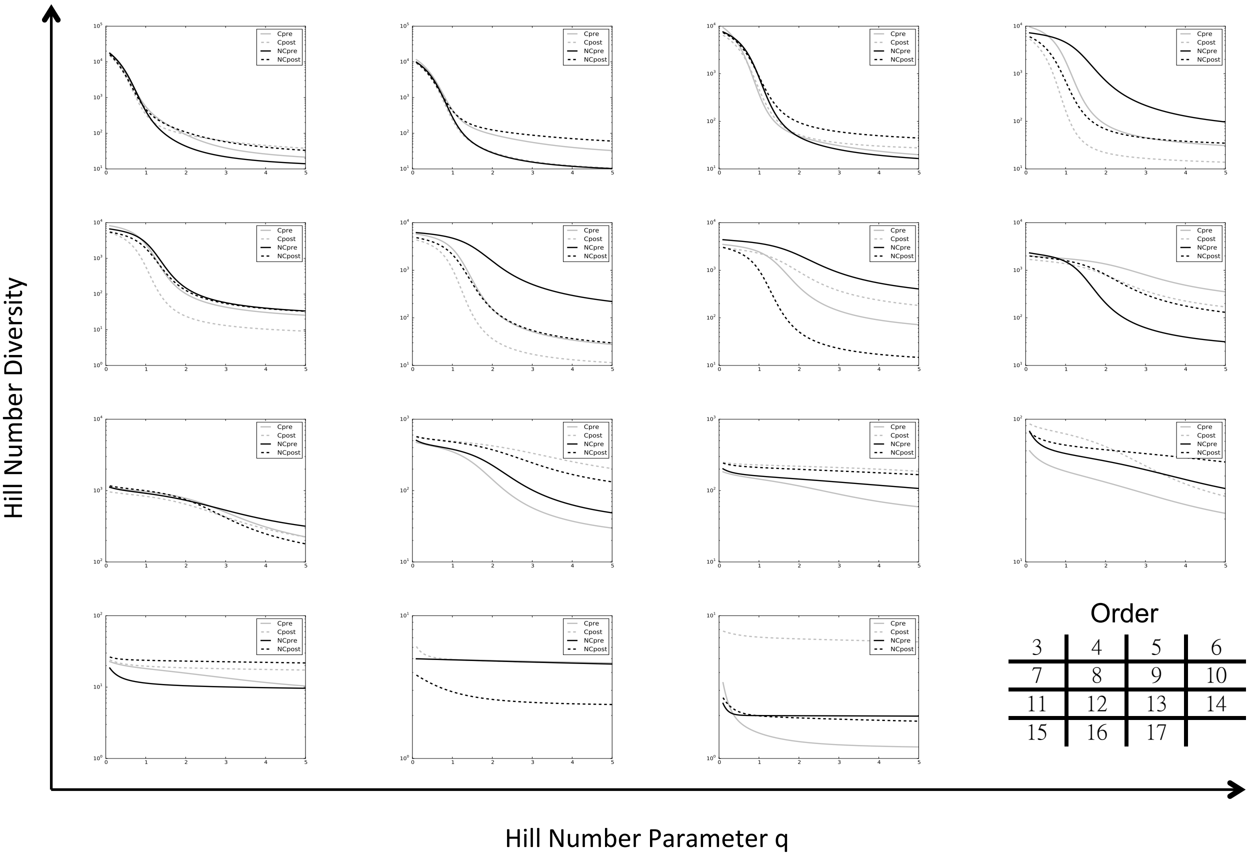
Hill Number Diversity
Hill Number Parameter q
Cpre
Cpost
NCpre
NCpost
Order
3 4 5 6
7 8 9 10
11 12 13 14
15 16 17

Supplementary Fig. 4

A

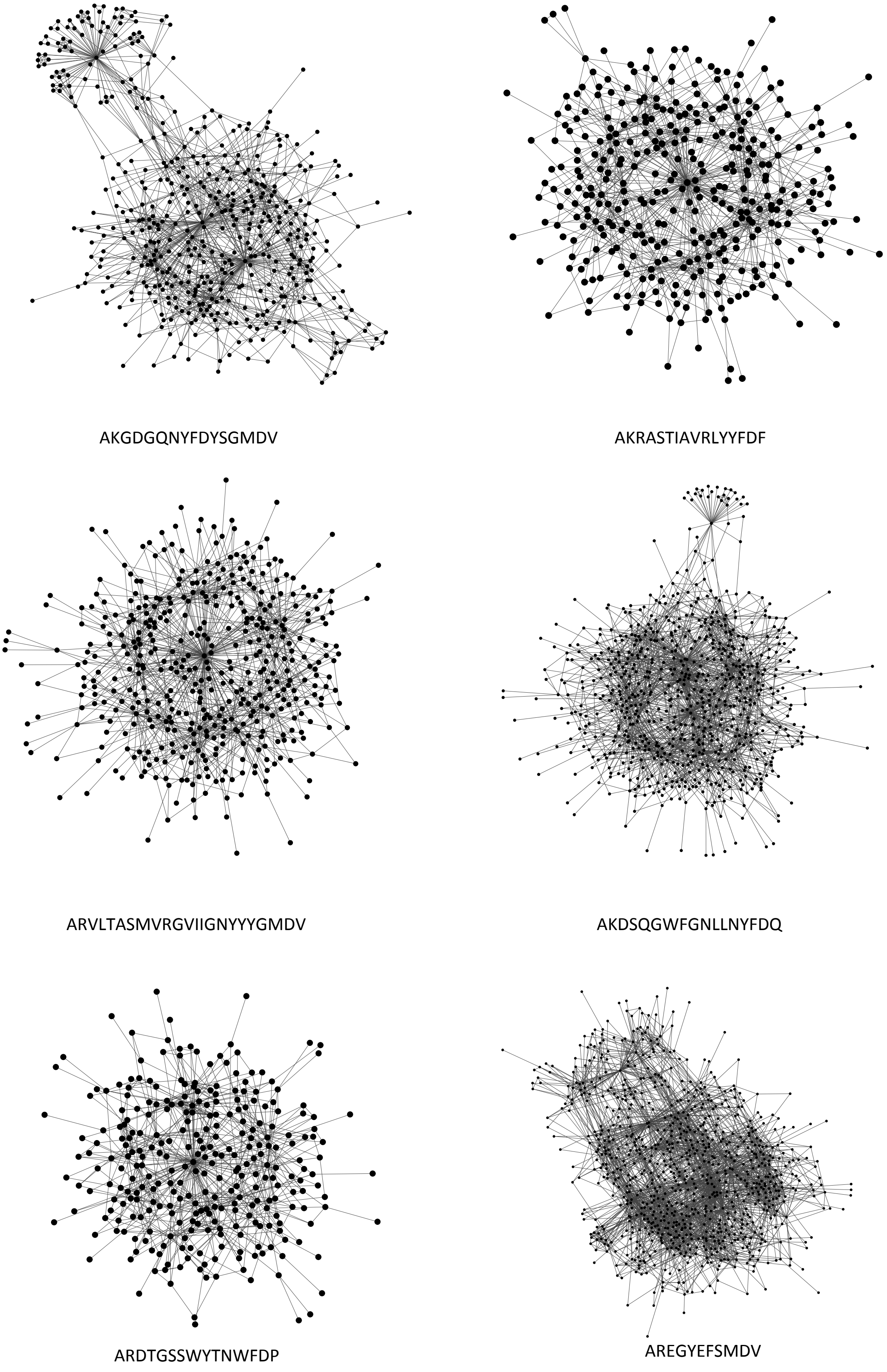

B

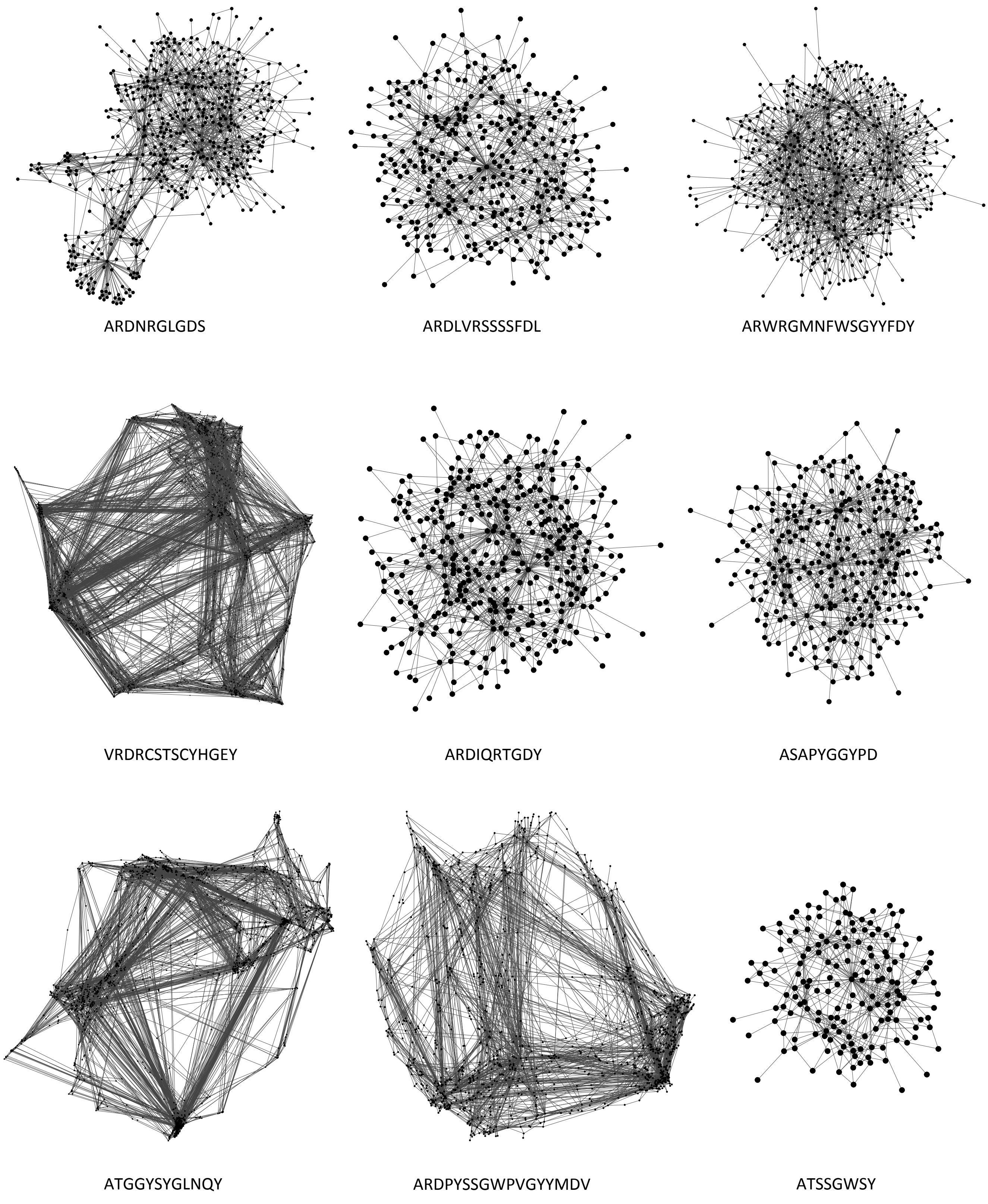
ARDNRGLGDS
ARDLVRSSSSFDL
ARWRGMNFWSGYYFDY
VRDRCSTSCYHGEY
ARDIQRTGDY
ASAPYGGYPD
ATGGYSYGLNQY
ARDPYSSGWPVGYYMDV
ATSSGWSY

Supplementary Fig. 5

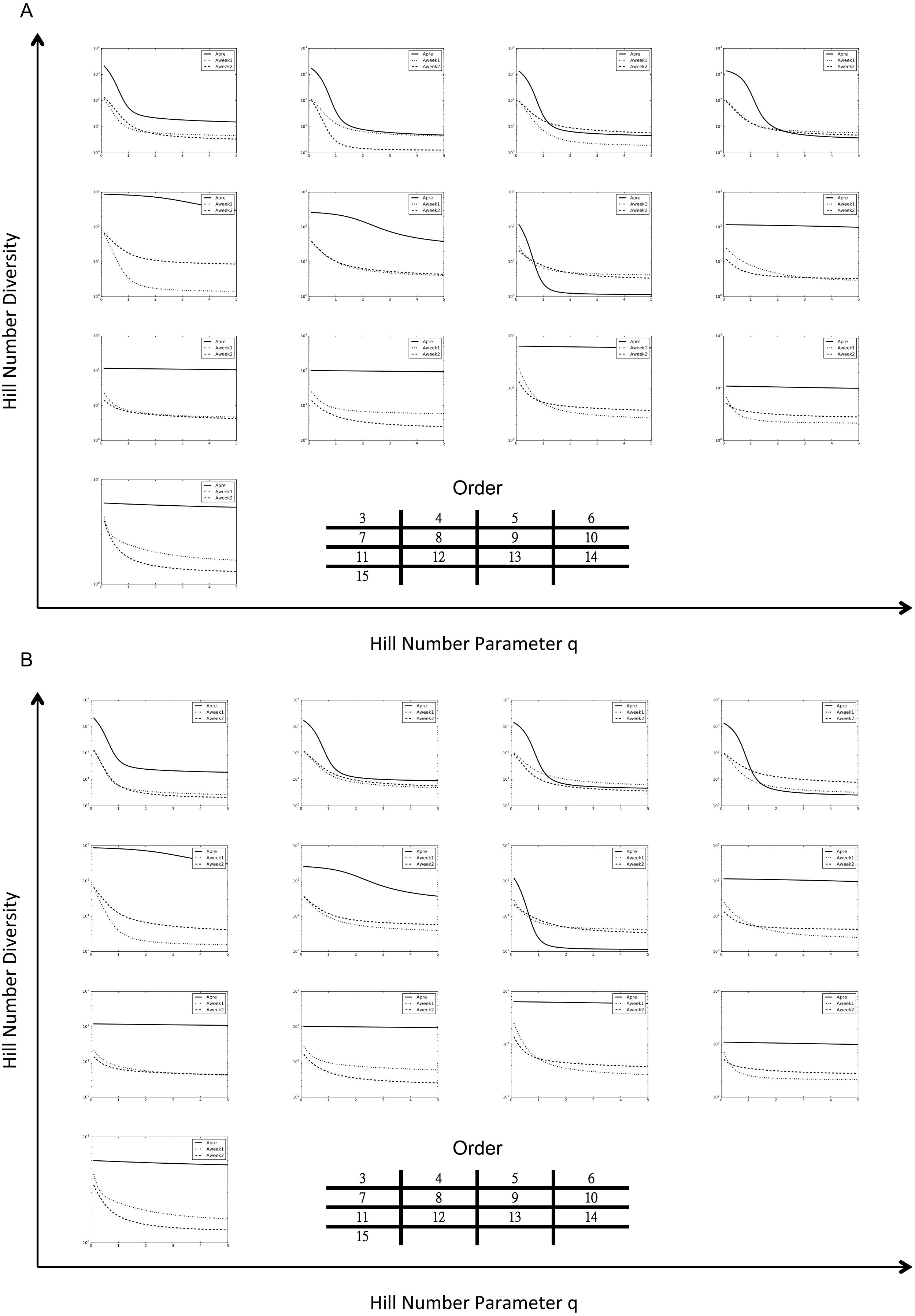

C

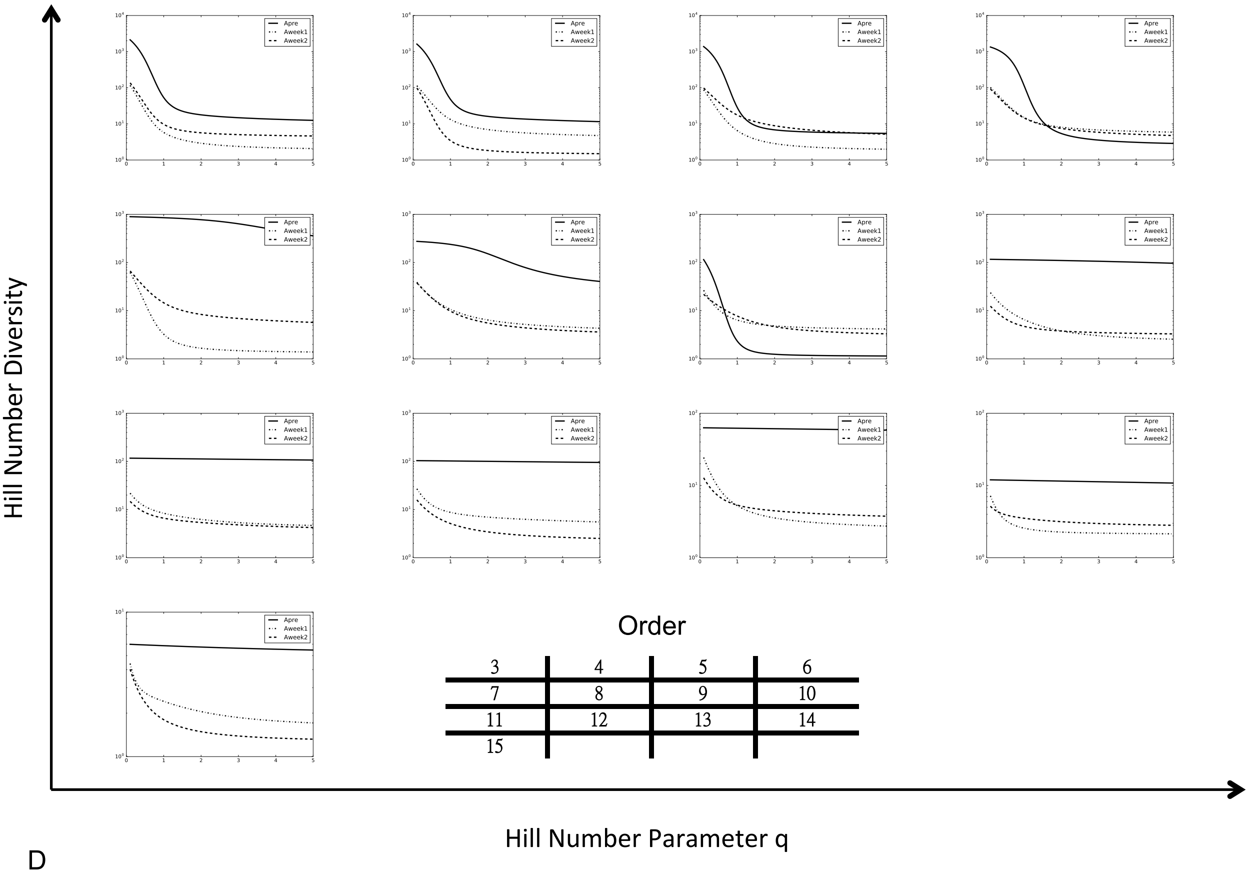
Hill Number Diversity
Hill Number Parameter q
Apre
Aweek1
Aweek2
Order
3 4 5 6
7 8 9 10
11 12 13 14
15

D

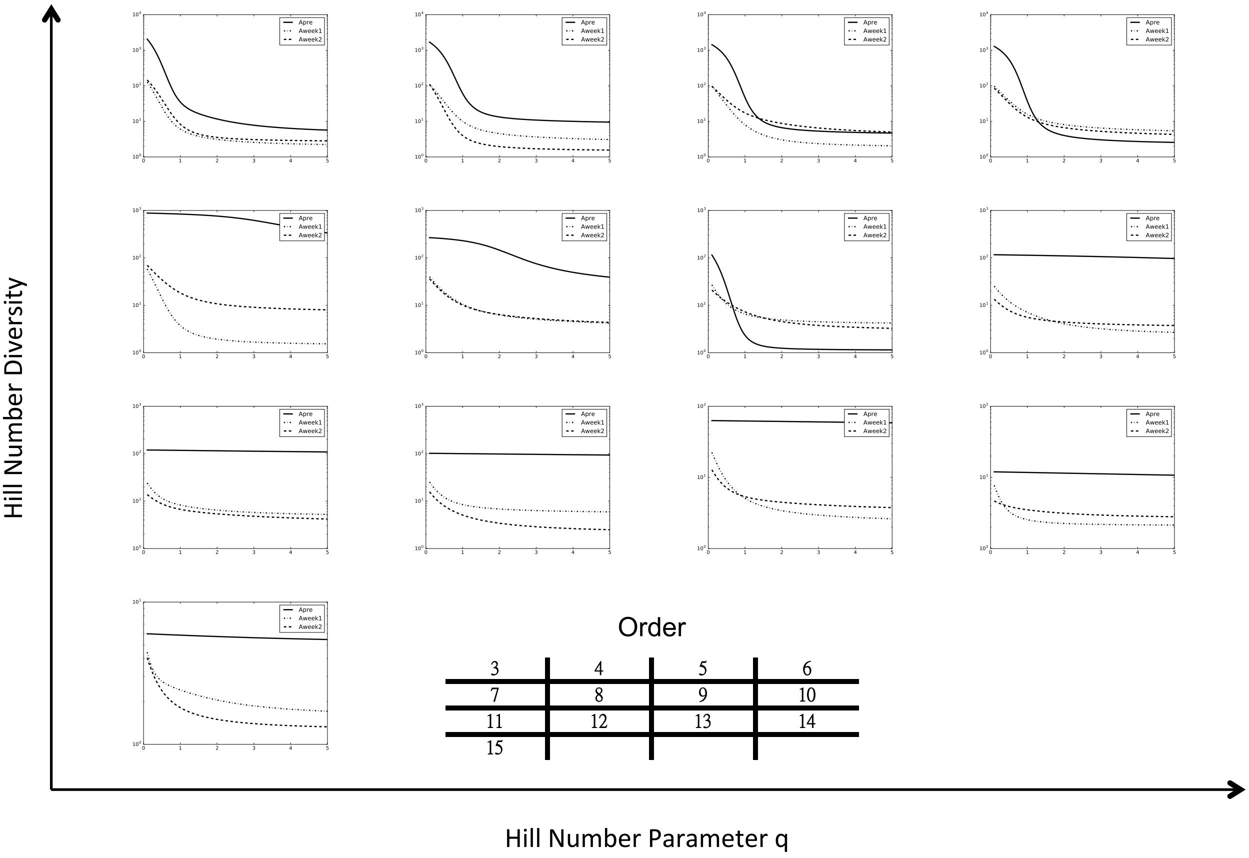
Hill Number Diversity
Hill Number Parameter q
Apre
Aweek1
Aweek2
Order
3 4 5 6
7 8 9 10
11 12 13 14
15

E

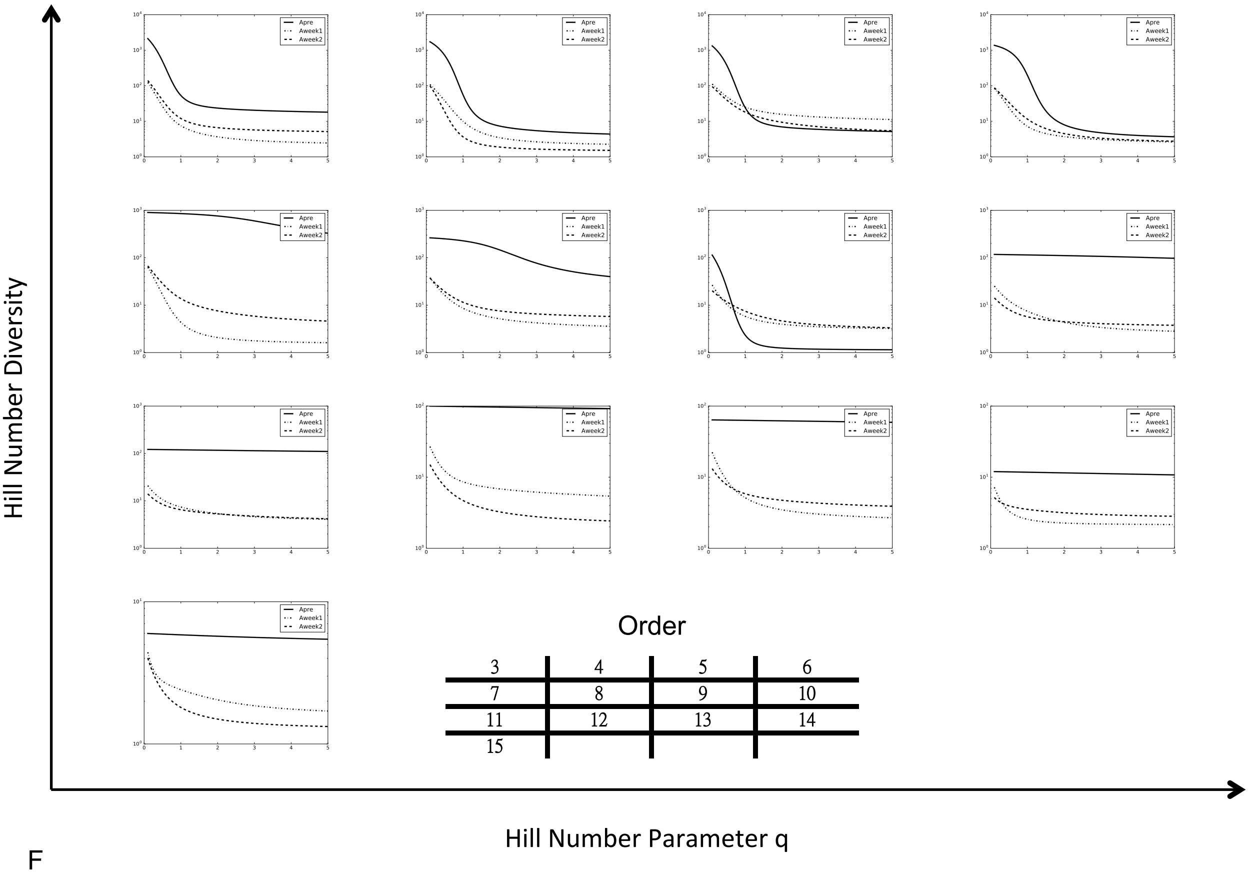
Hill Number Diversity
Apre
Aweek1
Aweek2
Order
3 4 5 6
7 8 9 10
11 12 13 14
15
Hill Number Parameter q

F

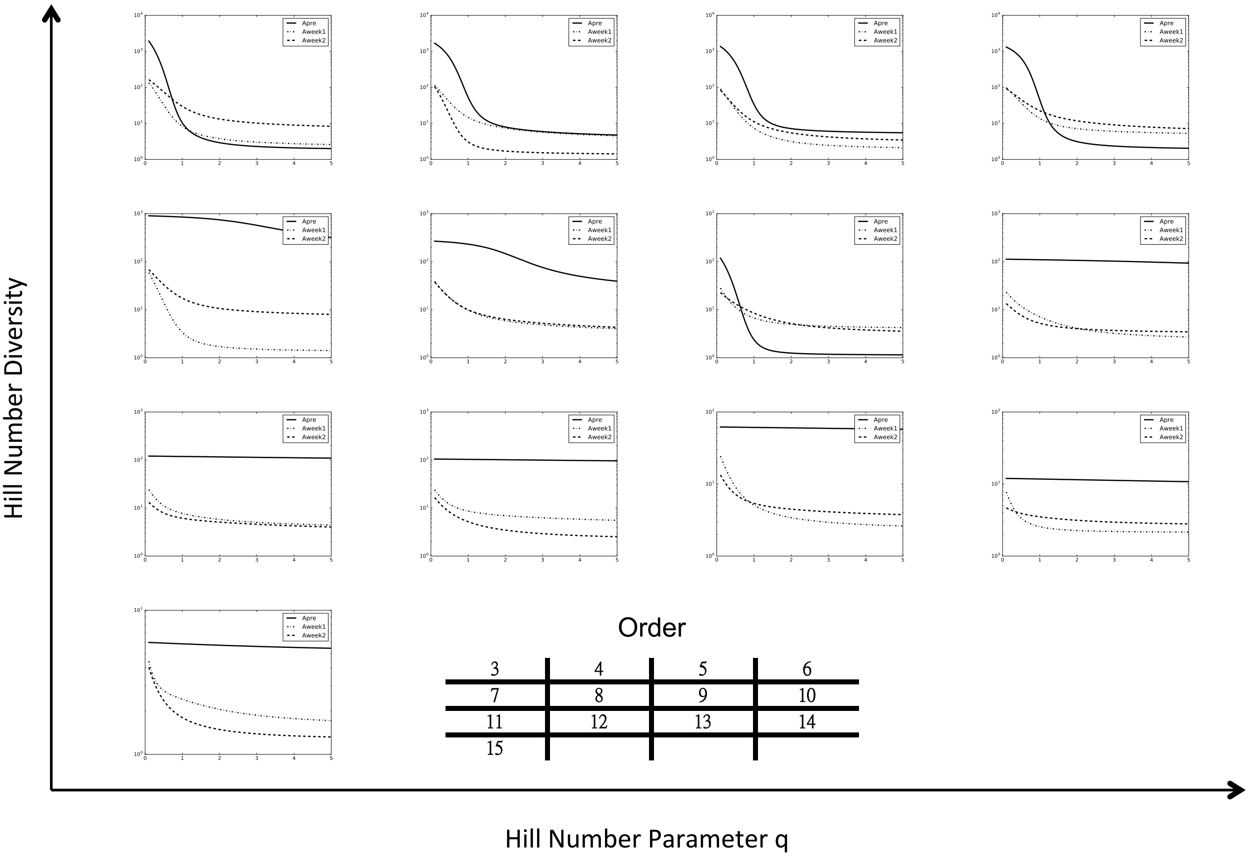
Hill Number Diversity
Apre
Aweek1
Aweek2
Order
3 4 5 6
7 8 9 10
11 12 13 14
15
Hill Number Parameter q

G

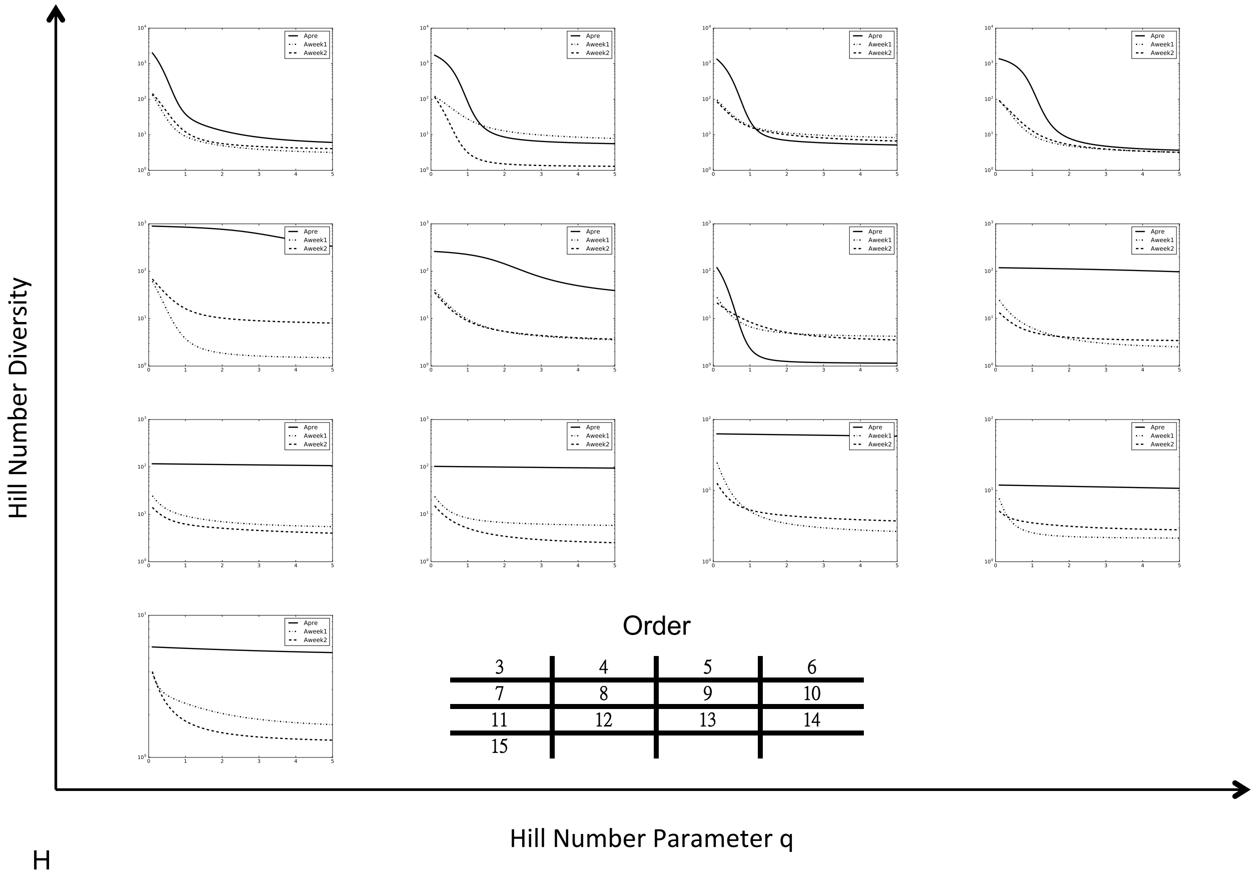
Hill Number Diversity
Apre
Aweek1
Aweek2
Order
3 4 5 6
7 8 9 10
11 12 13 14
15
Hill Number Parameter q

H

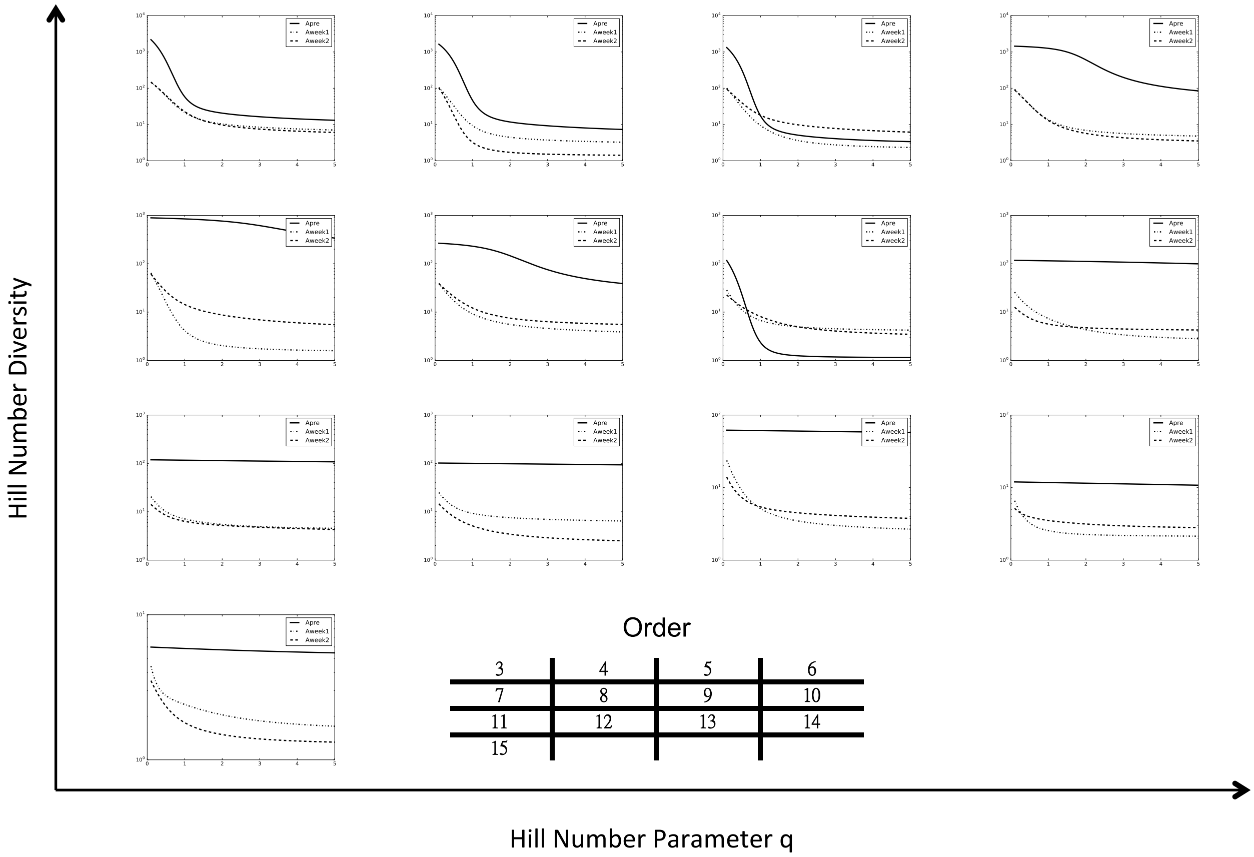
Hill Number Diversity
Apre
Aweek1
Aweek2
Order
3 4 5 6
7 8 9 10
11 12 13 14
15
Hill Number Parameter q

I

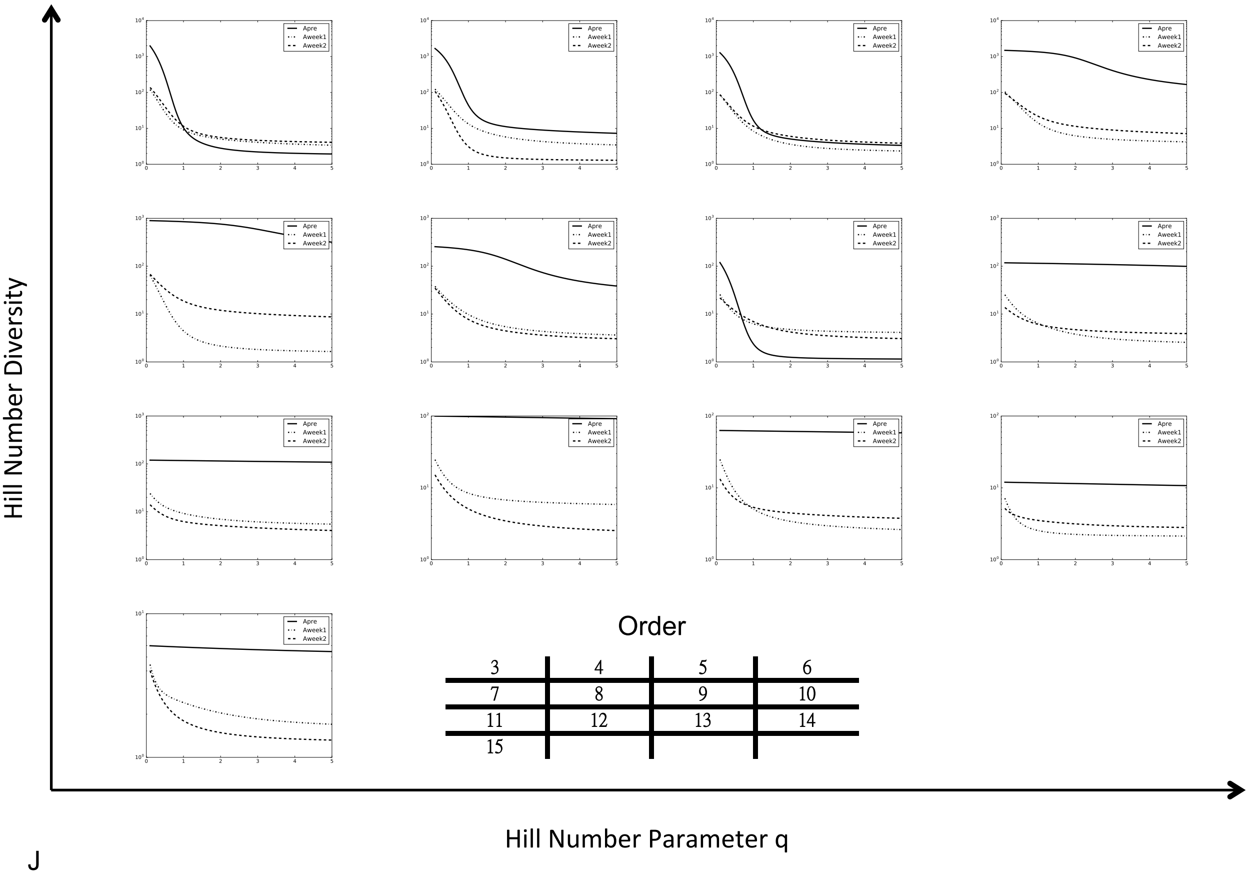
Hill Number Diversity
Hill Number Parameter q
Apre
Aweek1
Aweek2
Order
3 4 5 6
7 8 9 10
11 12 13 14
15

J

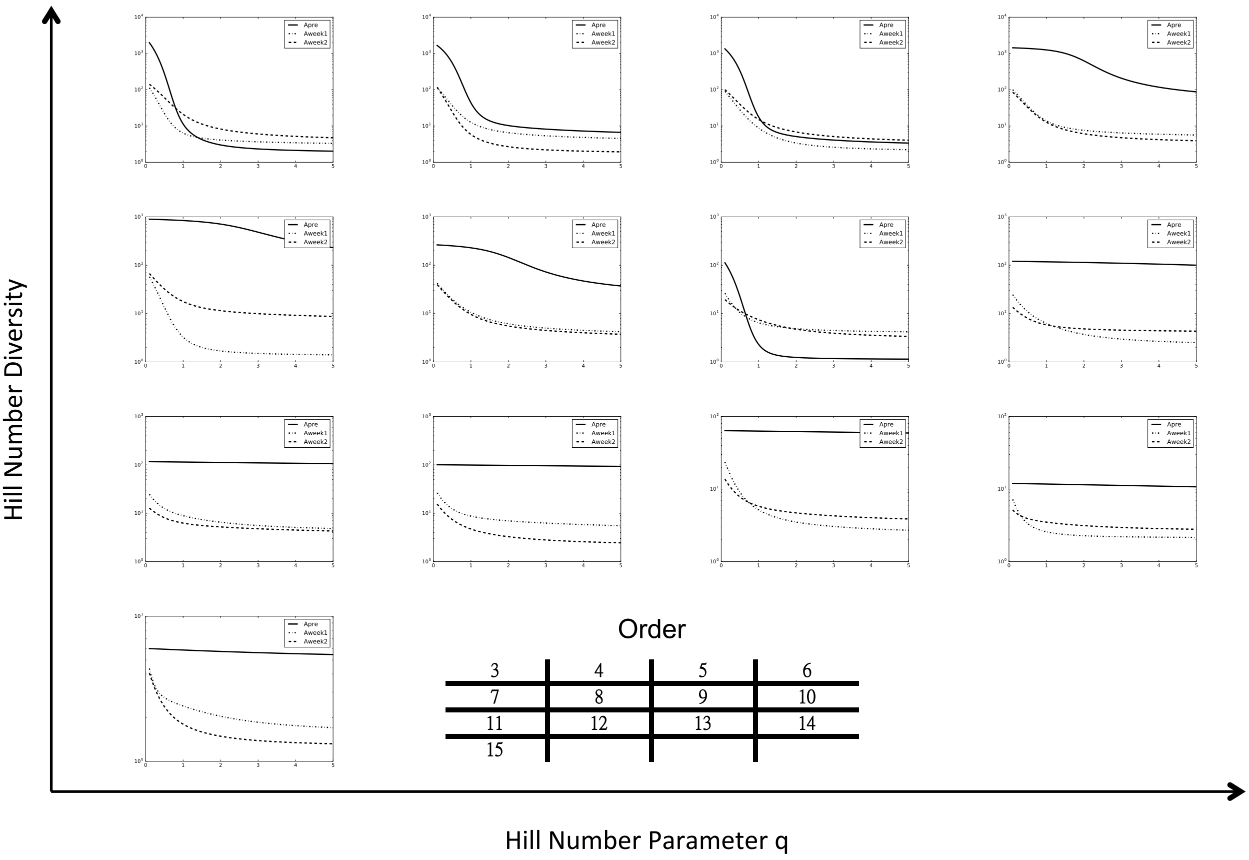
Hill Number Diversity
Hill Number Parameter q
Apre
Aweek1
Aweek2
Order
3 4 5 6
7 8 9 10
11 12 13 14
15

Supplementary Table 1

Sequence	Description
GAGTTCCACGACACCGTCACC	Primer for reverse transcription
GTGACTGGAGTTCAGACGTGTGCTCTTCCGATCTCCAGGGGGAAGACCGATGG	Primers paired with V-primers
GTGACTGGAGTTCAGACGTGTGCTCTTCCGATCTCCAGGGGGAAGACGGATGG	
ACACTCTTTCCCTACACGACGCTCTTCCGATCTACAGCCTACATGGAGCTGAGCA	V-primers
ACACTCTTTCCCTACACGACGCTCTTCCGATCTACATCCACGAGCACAGCCTACAT	
ACACTCTTTCCCTACACGACGCTCTTCCGATCTCACAGACCTGAGCAGCCTGAC	
ACACTCTTTCCCTACACGACGCTCTTCCGATCTGACACGTCCACGAGCACAGTCTA	
ACACTCTTTCCCTACACGACGCTCTTCCGATCTCCAAAAACCAGGTGGTCCTTACA	
ACACTCTTTCCCTACACGACGCTCTTCCGATCTCTCCAAAAGCCAGGTGGTCCT	
ACACTCTTTCCCTACACGACGCTCTTCCGATCTAACAGCCTGAGAGCCGAGGAC	
ACACTCTTTCCCTACACGACGCTCTTCCGATCTCGCCAAGAACTCCCTGTATCTG	
ACACTCTTTCCCTACACGACGCTCTTCCGATCTTGCCAAGAACTCCTTGTATCTTCA	
ACACTCTTTCCCTACACGACGCTCTTCCGATCTCACGCTGTATCTGCAAATGAAC	
ACACTCTTTCCCTACACGACGCTCTTCCGATCTAAAACACGCTGTATCTGCAAATGAT	
ACACTCTTTCCCTACACGACGCTCTTCCGATCTGAACAGACGGAGAGCCGAGGA	
ACACTCTTTCCCTACACGACGCTCTTCCGATCTTTCCTGTATCAGCAAATGAACAGC	
ACACTCTTTCCCTACACGACGCTCTTCCGATCTAGTCTGAGAGCCGAGGACACG	
ACACTCTTTCCCTACACGACGCTCTTCCGATCTGCCAAGAACACACTTCATCTGCAA	
ACACTCTTTCCCTACACGACGCTCTTCCGATCTGAACAGGCTGTATCTGCAAATGAAC	
ACACTCTTTCCCTACACGACGCTCTTCCGATCTCCAAGAACACGCTGTATCTGCAA	
ACACTCTTTCCCTACACGACGCTCTTCCGATCTAATAGCCTGAGGGCCGAGGAC	
ACACTCTTTCCCTACACGACGCTCTTCCGATCTTTCAAATGAACAACCTGAGAGCTGA	
ACACTCTTTCCCTACACGACGCTCTTCCGATCTCAAGAACACGCTGCATCTTCAAA	
ACACTCTTTCCCTACACGACGCTCTTCCGATCTGCAAAAACTCCCTGTATCTGCAA	
ACACTCTTTCCCTACACGACGCTCTTCCGATCTTGAACAGCCTGATAGCTGAGGA	
ACACTCTTTCCCTACACGACGCTCTTCCGATCTTGAACAGCCTGAGAGACGAGGA	
ACACTCTTTCCCTACACGACGCTCTTCCGATCTTCCAAAAGCATCGCCTATCTGC	
ACACTCTTTCCCTACACGACGCTCTTCCGATCTCCAAGAACACGCTGTATCTTCAAATG	
ACACTCTTTCCCTACACGACGCTCTTCCGATCTGACAATTCCAAGAACACGCTGTAT	
ACACTCTTTCCCTACACGACGCTCTTCCGATCTAAGAACTCACTGTATCTGCAAATGAAC	
ACACTCTTTCCCTACACGACGCTCTTCCGATCTCGGCGTATCTGCAAATGAACA	
ACACTCTTTCCCTACACGACGCTCTTCCGATCTGCCAAGAACACGCTGTATCTGC	
ACACTCTTTCCCTACACGACGCTCTTCCGATCTTCCCTGAAGCTGAGCTCTGTGAC	
ACACTCTTTCCCTACACGACGCTCTTCCGATCTCCTGAAGCCGAGCTCTGTGAC	
ACACTCTTTCCCTACACGACGCTCTTCCGATCTTCAGTAGACACGTCCAAGAACCAG	
ACACTCTTTCCCTACACGACGCTCTTCCGATCTACGTCTAAGAACCAGTTCTCCCTGA	
ACACTCTTTCCCTACACGACGCTCTTCCGATCTACGTCCAAGAACCAGTTCTACCTGA	
ACACTCTTTCCCTACACGACGCTCTTCCGATCTCCTGAAGCTGAGCTCTGTGACC	
ACACTCTTTCCCTACACGACGCTCTTCCGATCTTGCCTACCTGCAGTGGAGCAG	
ACACTCTTTCCCTACACGACGCTCTTCCGATCTTCCCTGCAGCTGAACTCTGTG	
ACACTCTTTCCCTACACGACGCTCTTCCGATCTCCTCTGTCAGCACGGCATATC	
ACACTCTTTCCCTACACGACGCTCTTCCGATCTCACCTCTGTCAGCATGGCATATC	
ACACTCTTTCCCTACACGACGCTCTTCCGATCTCAGCACGGCGTGTCTTCAGAT	
ACACTCTTTCCCTACACGACGCTCTTCCGATCTTCTGCCAGCACAGCATACCTG	
AATGATACGGCGACCACCGAGATCTACACCCTGCGAACACTCTTTCCCTACACGACGCTCTT	P5 primers with indices 1-10
AATGATACGGCGACCACCGAGATCTACACTGCAGAGACACTCTTTCCCTACACGACGCTCTT	
AATGATACGGCGACCACCGAGATCTACACACCTAGGACACTCTTTCCCTACACGACGCTCTT	
AATGATACGGCGACCACCGAGATCTACACTTGATCCACACTCTTTCCCTACACGACGCTCTT	
AATGATACGGCGACCACCGAGATCTACACATCTTGCACACTCTTTCCCTACACGACGCTCTT	
AATGATACGGCGACCACCGAGATCTACACTCTCCATACACTCTTTCCCTACACGACGCTCTT	
AATGATACGGCGACCACCGAGATCTACACCATCGAGACACTCTTTCCCTACACGACGCTCTT	
AATGATACGGCGACCACCGAGATCTACACTTCGAGCACACTCTTTCCCTACACGACGCTCTT	
AATGATACGGCGACCACCGAGATCTACACAGTTGGTACACTCTTTCCCTACACGACGCTCTT	
AATGATACGGCGACCACCGAGATCTACACGTACCGGACACTCTTTCCCTACACGACGCTCTT	
CAAGCAGAAGACGGCATACGAGATGAAGATTGTGACTGGAGTTCAGACGTGT	P7 primers with indices 1-10
CAAGCAGAAGACGGCATACGAGATCGTTGGTGTGACTGGAGTTCAGACGTGT	
CAAGCAGAAGACGGCATACGAGATGCCATCTGTGACTGGAGTTCAGACGTGT	
CAAGCAGAAGACGGCATACGAGATAACCTGGGTGACTGGAGTTCAGACGTGT	
CAAGCAGAAGACGGCATACGAGATCTAACGGGTGACTGGAGTTCAGACGTGT	
CAAGCAGAAGACGGCATACGAGATAGAGGCGGTGACTGGAGTTCAGACGTGT	
CAAGCAGAAGACGGCATACGAGATCCGCAAGGTGACTGGAGTTCAGACGTGT	
CAAGCAGAAGACGGCATACGAGATCTCCGCCGTGACTGGAGTTCAGACGTGT	
CAAGCAGAAGACGGCATACGAGATACGTCCAGTGACTGGAGTTCAGACGTGT	
CAAGCAGAAGACGGCATACGAGATCATGGTTGTGACTGGAGTTCAGACGTGT	

Supplementary Table 2

	$F1C_{pre}$	$F1C_{post}$	$F2C_{pre}$	$F2C_{post}$	$F3C_{pre}$	$F3C_{post}$	$F4C_{pre}$	$F4C_{post}$	$F1NC_{pre}$	$F1NC_{post}$	$F2NC_{pre}$	$F2NC_{post}$	$F3NC_{pre}$	$F3NC_{post}$	$F4NC_{pre}$	$F4NC_{post}$
$F1C_{pre}$	-	0.9520422	0.9973684	0.9999285	0.9975994	0.9992634	0.9998889	0.9998626	0.9998698	0.9987032	0.9980289	0.9996612	0.9999916	0.9999925	0.9998939	0.9994598
$F1C_{post}$	0.9520422	-	0.9997043	0.9998895	0.9999649	0.9985481	0.9999171	0.9999760	0.9999816	0.9999852	0.9999693	0.9999869	0.9999891	0.9999996	0.9999695	0.9999952
$F2C_{pre}$	0.9973684	0.9997043	-	0.9564328	0.9999978	0.9998627	0.9962231	0.9996804	0.9993394	0.9986972	0.9983587	0.9986251	0.9999953	0.9999936	0.9999018	0.9999466
$F2C_{post}$	0.9999285	0.9998895	0.9564328	-	0.9999988	0.9998897	0.9991903	0.9986955	0.9999759	0.9999754	0.9999910	0.9999994	0.9998929	0.9999518	0.9999912	0.9999959
$F3C_{pre}$	0.9975994	0.9999649	0.9999978	0.9999988	-	0.9600126	0.9999774	0.9998113	0.9999822	0.9999474	0.9999917	0.9999999	0.9999366	0.9998646	0.9974071	0.9999963
$F3C_{post}$	0.9992634	0.9985481	0.9998627	0.9998897	0.9600126	-	0.9999432	0.9999724	0.9987360	0.9995761	0.9998532	0.9999522	0.9998976	0.9999813	0.9999993	0.9999992
$F4C_{pre}$	0.9998889	0.9999171	0.9962231	0.9991903	0.9999774	0.9999432	-	0.9498923	0.9999806	0.9999953	0.9999624	0.9999958	0.9999611	0.9999377	0.9938726	0.9997232
$F4C_{post}$	0.9998626	0.9999760	0.9996804	0.9986955	0.9998113	0.9999724	0.9498923	-	0.9999814	0.9999089	0.9999811	0.9999958	0.9999440	0.9999465	0.9946237	0.9996588
$F1NC_{pre}$	0.9998698	0.9999816	0.9993394	0.9999759	0.9999822	0.9987360	0.9999806	0.9999814	-	0.9125702	0.9996909	0.9999769	0.9997883	0.9994165	0.9999832	0.9999794
$F1NC_{post}$	0.9987032	0.9999852	0.9986972	0.9999754	0.9999474	0.9995761	0.9999953	0.9999089	0.9125702	-	0.9995235	0.9998812	0.9997151	0.9994518	0.9999662	0.9999668
$F2NC_{pre}$	0.9980289	0.9999693	0.9983587	0.9999910	0.9999917	0.9998532	0.9999624	0.9999811	0.9996909	0.9995235	-	0.9165187	0.9998643	0.9996570	0.9962939	0.9999327
$F2NC_{post}$	0.9996612	0.9999869	0.9986251	0.9999994	0.9999999	0.9999522	0.9999958	0.9999958	0.9999769	0.9998812	0.9165187	-	0.9998138	0.9999755	0.9993115	0.9948752
$F3NC_{pre}$	0.9999916	0.9999891	0.9999953	0.9998929	0.9999366	0.9998976	0.9999611	0.9999440	0.9997883	0.9997151	0.9998643	0.9998138	-	0.9563739	0.9999581	0.9999984
$F3NC_{post}$	0.9999925	0.9999996	0.9999936	0.9999518	0.9998646	0.9999813	0.9999377	0.9999465	0.9994165	0.9994518	0.9996570	0.9999755	0.9563739	-	0.9999487	0.9999995
$F4NC_{pre}$	0.9998939	0.9999695	0.9999018	0.9999912	0.9974071	0.9999993	0.9938726	0.9946237	0.9999832	0.9999662	0.9962939	0.9993115	0.9999581	0.9999487	-	0.9945914
$F4NC_{post}$	0.9994598	0.9999952	0.9999466	0.9999959	0.9999963	0.9999992	0.9997232	0.9996588	0.9999794	0.9999668	0.9999327	0.9948752	0.9999984	0.9999995	0.9945914	-

Similar   Dissimilar

Supplementary Table 3

Order		3	4	5	6	7	8	9	10	11	12	13	14	15	16	17
Carriers		-	-	±	-	-	-	+	-	-	+	+	+	+	±	+
Rarefaction	1	-	-	+	-	-	-	+	-	-	+	+	+	+	±	+
	2	+	-	-	-	-	-	+	-	-	+	+	+	+	±	+
	3	-	-	-	-	-	-	+	-	-	+	+	+	+	±	+
	4	-	-	+	-	-	-	+	-	-	+	+	+	+	±	+
	5	-	-	+	-	-	-	+	-	-	+	+	+	+	±	+
	6	-	+	-	-	-	-	+	-	-	+	+	+	+	±	+
	7	-	-	-	-	-	-	+	-	-	+	+	+	+	±	+
	8	-	-	+	-	-	-	+	-	-	+	+	+	+	±	+
	9	-	-	-	-	-	-	+	-	-	+	+	+	+	±	+
	10	+	-	+	-	-	-	+	-	-	+	+	+	+	±	+
Non-carriers		+	+	+	-	-	-	-	+	-	+	+	+	+	-	-
Rarefaction	1	+	+	+	-	-	-	-	+	-	+	+	+	+	-	-
	2	+	+	+	-	-	-	-	+	-	+	+	+	+	-	-
	3	+	+	+	-	-	-	-	+	-	+	+	+	+	-	-
	4	+	+	+	-	-	-	-	+	-	+	+	+	+	-	-
	5	+	+	+	-	-	-	-	+	-	+	+	+	+	-	-
	6	+	-	+	-	-	-	-	+	-	+	+	+	+	-	-
	7	+	+	+	-	-	-	-	+	-	+	+	+	+	-	-
	8	+	+	+	-	-	-	-	+	-	+	+	+	+	-	-
	9	+	+	+	-	-	-	-	+	-	+	+	+	+	-	-
	10	+	+	+	-	-	-	-	+	-	+	+	+	+	-	-

Supplementary Table 4

Cluster Rank		1	2	3	4	5	6	7	8	9	10	11	12	13	14	15	16	17	18	19	20	21	22	23	24	25
SVC Coefficients in 10 Rarefaction Datasets	1	0.0	24.6	0.0	24.6	0.0	0.0	0.0	23.9	0.0	0.0	35.6	0.0	0.0	0.0	0.0	0.0	0.0	0.0	0.0	0.0	0.0	0.0	0.0	0.0	0.0
	2	0.0	24.3	0.0	23.5	0.0	24.2	0.0	0.0	0.0	0.0	0.0	0.0	0.0	0.0	0.0	0.0	0.0	0.0	0.0	0.0	28.3	-0.7	0.0	0.0	0.0
	3	0.0	24.4	0.0	0.0	0.0	24.7	0.0	0.0	0.0	0.0	0.0	0.0	0.0	-0.6	25.6	35.6	0.0	0.0	0.0	0.0	0.0	0.0	0.0	0.0	0.0
	4	0.0	0.0	24.2	0.0	0.0	0.0	24.3	21.7	0.0	0.0	0.0	0.0	0.0	0.0	0.0	28.4	0.0	0.0	0.0	0.0	0.0	0.0	0.0	0.0	0.0
	5	0.0	24.3	21.9	0.0	24.2	0.0	0.0	0.0	0.0	0.0	0.0	0.0	0.0	0.0	0.0	0.0	0.0	0.0	0.0	0.0	0.0	0.0	28.2	0.0	0.0
	6	24.1	0.0	0.0	0.0	21.6	0.0	24.7	0.0	0.0	0.0	0.0	0.0	0.0	0.0	0.0	35.7	0.0	0.0	0.0	0.0	-0.5	0.0	0.0	0.0	0.0
	7	0.0	24.5	0.0	24.2	0.0	0.0	23.8	0.0	0.0	0.0	28.4	0.0	0.0	0.0	0.0	0.0	0.0	0.0	0.0	0.0	0.0	0.0	0.0	0.0	0.0
	8	24.4	0.0	0.0	0.0	0.0	21.8	0.0	0.0	24.6	0.0	0.0	0.0	0.0	0.0	0.0	0.0	0.0	35.8	0.0	0.0	0.0	0.0	0.0	0.0	0.0
	9	24.4	0.0	0.0	23.8	0.0	24.9	0.0	0.0	0.0	0.0	0.0	35.6	0.0	-0.3	0.0	0.0	0.0	0.0	0.0	0.0	0.0	0.0	0.0	0.0	0.0
	10	0.0	0.0	0.0	0.0	0.0	24.2	0.0	0.0	23.9	26.3	0.0	28.5	0.0	0.0	0.0	0.0	0.0	0.0	0.0	0.0	0.0	0.0	0.0	0.0	0.0
LR Coefficients in 10 Rarefaction Datasets	1	0.0	27.8	0.0	61.4	0.0	0.0	0.0	43.6	0.0	0.0	79.9	15.0	0.0	0.0	0.0	0.0	0.0	21.2	0.0	0.0	0.0	0.0	0.0	0.0	0.0
	2	0.0	40.8	0.0	42.4	0.0	62.1	0.0	0.0	0.0	16.2	0.0	19.9	0.0	0.0	0.0	0.0	0.0	0.0	0.0	2.3	71.3	-1.1	0.0	0.0	0.0
	3	0.0	62.7	0.0	0.0	0.0	62.7	0.0	0.0	0.0	0.0	0.0	0.0	0.0	0.0	42.3	83.8	0.0	0.0	0.0	0.0	0.0	0.0	0.0	0.0	0.0
	4	0.0	0.0	48.8	0.0	0.0	0.0	62.7	50.5	14.1	0.0	0.0	0.0	0.0	0.0	8.4	72.1	0.0	0.0	0.0	0.0	0.0	0.0	0.0	0.0	0.0
	5	0.0	24.1	33.6	0.0	61.8	0.0	0.0	12.7	25.2	0.0	0.0	20.2	0.0	0.0	0.0	0.0	0.0	0.0	4.4	0.0	0.0	0.0	70.9	0.0	0.0
	6	44.4	0.0	0.0	14.7	38.0	0.0	61.9	0.0	0.0	0.0	16.5	0.0	0.0	0.0	0.0	82.2	0.0	0.0	0.0	0.0	-1.1	0.0	0.0	0.0	0.0
	7	0.0	31.4	0.0	60.9	0.0	6.3	47.9	5.3	0.0	31.8	70.4	0.0	0.0	0.0	0.0	0.0	0.0	0.0	0.0	0.0	0.0	0.0	0.0	0.0	0.0
	8	32.4	0.0	0.0	0.0	0.0	30.2	0.0	0.0	61.7	0.0	27.8	22.3	0.0	0.0	0.0	0.0	0.0	81.8	0.0	0.0	0.0	9.4	0.0	0.0	0.0
	9	32.7	0.0	1.1	46.9	0.0	63.2	0.0	0.0	0.0	0.0	9.8	83.6	0.6	0.0	25.4	0.0	5.3	0.0	0.0	0.0	0.0	0.0	0.0	0.0	0.0
	10	0.0	0.0	0.0	0.0	30.8	62.3	0.0	0.0	62.2	31.2	0.0	71.6	0.0	0.0	0.0	5.6	0.0	0.0	0.0	0.0	0.0	0.0	0.0	0.0	0.0

Cluster Rank		26	27	28	29	30	31	32	33	34	35	36	37	38	39	40	41	42	43	44	45	46	47	48	49	50
SVC Coefficients in 10 Rarefaction Datasets	1	0.0	0.0	0.0	0.0	0.0	0.0	0.0	0.0	0.0	0.0	0.0	0.0	0.0	0.0	0.0	0.0	0.0	0.0	0.0	0.0	0.0	0.0	0.0	0.0	0.0
	2	0.0	0.0	0.0	0.0	0.0	0.0	0.0	0.0	0.0	0.0	0.0	0.0	0.0	0.0	0.0	0.0	0.0	0.0	0.0	0.0	0.0	0.0	0.0	0.0	0.0
	3	0.0	0.0	0.0	0.0	0.0	0.0	0.0	0.0	0.0	0.0	0.0	0.0	0.0	0.0	0.0	0.0	0.0	0.0	0.0	0.0	0.0	0.0	0.0	0.0	0.0
	4	0.0	0.0	0.0	0.0	0.0	0.0	0.0	0.0	0.0	0.0	0.0	0.0	0.0	0.0	0.0	0.0	0.0	0.0	0.0	0.0	0.0	0.0	0.0	0.0	0.0
	5	0.0	0.0	0.0	0.0	0.0	0.0	0.0	0.0	0.0	0.0	0.0	0.0	0.0	0.0	0.0	0.0	0.0	0.0	0.0	0.0	0.0	0.0	0.0	0.0	0.0
	6	0.0	0.0	0.0	0.0	0.0	0.0	0.0	0.0	0.0	0.0	0.0	0.0	0.0	0.0	0.0	0.0	0.0	0.0	0.0	0.0	0.0	0.0	0.0	0.0	0.0
	7	0.0	0.0	0.0	0.0	0.0	0.0	0.0	0.0	0.0	0.0	0.0	0.0	0.0	0.0	0.0	0.0	0.0	0.0	0.0	0.0	0.0	0.0	0.0	0.0	0.0
	8	0.0	0.0	0.0	0.0	0.0	0.0	0.0	0.0	0.0	0.0	0.0	0.0	0.0	0.0	0.0	0.0	0.0	0.0	0.0	0.0	0.0	0.0	0.0	0.0	0.0
	9	0.0	0.0	0.0	0.0	0.0	0.0	0.0	0.0	0.0	0.0	0.0	0.0	0.0	0.0	0.0	0.0	0.0	0.0	0.0	0.0	0.0	0.0	0.0	0.0	0.0
	10	0.0	0.0	0.0	0.0	0.0	0.0	0.0	0.0	0.0	0.0	0.0	0.0	0.0	0.0	0.0	0.0	0.0	0.0	0.0	0.0	0.0	0.0	0.0	0.0	0.0
LR Coefficients in 10 Rarefaction Datasets	1	0.0	0.0	0.0	18.3	0.0	0.0	0.0	0.0	0.0	0.0	0.0	0.0	0.0	0.0	0.0	0.0	0.0	0.0	0.0	0.0	0.0	0.0	0.0	0.0	0.0
	2	0.0	0.0	0.0	0.0	0.0	0.0	0.0	0.0	0.0	3.3	0.0	0.0	0.0	0.0	0.0	0.0	0.0	0.0	0.0	0.0	0.0	0.0	0.0	0.0	0.0
	3	0.0	0.0	0.0	0.0	0.0	0.0	0.0	0.0	0.0	0.0	0.0	0.0	0.0	0.0	0.0	0.0	0.0	0.0	22.7	0.0	0.0	0.0	0.0	0.0	0.0
	4	0.0	0.0	0.0	0.0	0.0	0.0	0.0	0.0	0.0	0.0	0.0	0.0	0.0	0.0	0.0	0.0	0.0	0.0	0.0	0.0	0.0	0.0	0.0	0.0	0.0
	5	0.0	0.0	0.0	0.0	0.0	3.4	0.0	0.0	0.0	0.0	0.0	0.0	0.0	0.0	0.0	0.0	0.0	0.0	0.0	0.0	0.0	0.0	0.0	0.0	0.0
	6	0.0	0.0	0.0	0.0	2.6	0.0	0.0	0.0	0.0	0.0	0.0	0.0	0.0	0.0	0.0	0.0	0.0	0.0	0.0	0.0	0.0	0.0	0.0	0.0	0.0
	7	0.0	0.0	0.0	0.0	0.0	0.0	0.0	0.0	0.0	0.0	0.0	0.0	0.0	0.0	0.0	0.0	0.0	0.0	0.0	0.0	0.0	0.0	0.0	0.0	0.0
	8	0.0	0.0	0.0	0.0	0.0	0.0	0.0	0.0	0.0	0.0	0.0	0.0	0.0	0.0	0.0	0.0	0.0	0.0	0.0	0.0	0.0	0.0	0.0	0.0	0.0
	9	0.0	0.0	4.5	0.0	0.0	0.0	0.0	0.0	0.0	0.0	0.0	0.0	0.0	0.0	0.0	0.0	0.0	0.0	0.0	0.0	0.0	0.0	0.0	0.0	0.0
	10	0.0	0.0	0.0	0.0	0.0	0.0	0.0	0.0	0.0	0.0	0.0	0.0	0.0	0.0	0.0	0.0	0.0	0.0	0.0	0.0	0.0	0.0	0.0	0.0	0.0

Cluster Rank		51	52	53	54	55	56	57	58	59	60	61	62	63	64	65	66	67	68	69	70	71	72	73	74
SVC Coefficients in 10 Rarefaction Datasets	1	0.0	0.0	0.0	0.0	0.0	0.0	0.0	0.0	0.0	0.0	0.0	0.0	0.0	0.0	0.0	0.0	0.0	0.0	0.0	0.0	0.0	0.0	0.0	0.0
	2	0.0	0.0	0.0	0.0	0.0	0.0	0.0	0.0	0.0	0.0	0.0	0.0	0.0	0.0	0.0	0.0	0.0	0.0	0.0	0.0	0.0	0.0	0.0	0.0
	3	0.0	0.0	0.0	0.0	0.0	0.0	0.0	0.0	0.0	0.0	0.0	0.0	0.0	0.0	0.0	0.0	0.0	0.0	0.0	0.0	0.0	0.0	0.0	0.0
	4	0.0	0.0	0.0	0.0	0.0	0.0	0.0	0.0	0.0	0.0	0.0	0.0	0.0	0.0	0.0	0.0	0.0	0.0	0.0	0.0	0.0	0.0	0.0	0.0
	5	0.0	0.0	0.0	0.0	0.0	0.0	0.0	0.0	0.0	0.0	0.0	0.0	0.0	0.0	0.0	0.0	0.0	0.0	0.0	0.0	0.0	0.0	0.0	0.0
	6	0.0	0.0	0.0	0.0	0.0	0.0	0.0	0.0	0.0	0.0	0.0	0.0	0.0	0.0	0.0	0.0	0.0	0.0	0.0	0.0	0.0	0.0	0.0	0.0
	7	0.0	0.0	0.0	0.0	0.0	0.0	0.0	0.0	0.0	0.0	0.0	0.0	0.0	0.0	0.0	0.0	0.0	0.0	0.0	0.0	0.0	0.0	0.0	0.0
	8	0.0	0.0	0.0	0.0	0.0	0.0	0.0	0.0	0.0	0.0	0.0	0.0	0.0	0.0	0.0	0.0	0.0	0.0	0.0	0.0	0.0	0.0	0.0	0.0
	9	0.0	0.0	0.0	0.0	0.0	0.0	0.0	0.0	0.0	0.0	0.0	0.0	0.0	0.0	0.0	0.0	0.0	0.0	0.0	0.0	0.0	0.0	0.0	0.0
	10	0.0	0.0	0.0	0.0	0.0	0.0	0.0	0.0	0.0	0.0	0.0	0.0	0.0	0.0	0.0	0.0	0.0	0.0	0.0	0.0	0.0	0.0	0.0	0.0
LR Coefficients in 10 Rarefaction Datasets	1	0.0	0.0	0.0	0.0	0.0	0.0	0.0	0.0	0.0	0.0	0.0	0.0	0.0	0.0	0.0	0.0	0.0	0.0	0.0	0.0	0.0	0.0	0.0	0.0
	2	0.0	0.0	0.0	0.0	0.0	0.0	0.0	0.0	0.0	0.0	0.0	0.0	0.0	0.0	0.0	0.0	0.0	0.0	0.0	0.0	0.0	0.0	0.0	0.0
	3	0.0	0.0	0.0	0.0	0.0	0.0	0.0	0.0	0.0	0.0	0.0	0.0	0.0	0.0	0.0	0.0	0.0	0.0	0.0	0.0	0.0	0.0	0.0	0.0
	4	0.0	0.0	0.0	0.0	0.0	0.0	0.0	0.0	0.0	0.0	0.0	0.0	0.0	0.0	0.0	0.0	0.0	0.0	0.0	0.0	0.0	0.0	0.0	0.0
	5	0.0	0.0	0.0	0.0	0.0	0.0	0.0	0.0	0.0	0.0	0.0	0.0	0.0	0.0	0.0	0.0	0.0	0.0	0.0	0.0	0.0	0.0	0.0	0.0
	6	0.0	0.0	0.0	0.0	0.0	0.0	0.0	0.0	0.0	0.0	0.0	0.0	0.0	0.0	0.0	0.0	0.0	0.0	0.0	0.0	0.0	0.0	0.0	0.0
	7	0.0	0.0	0.0	0.0	0.0	0.0	0.0	0.0	0.0	0.0	0.0	0.0	0.0	0.0	0.0	0.0	0.0	0.0	0.0	0.0	0.0	0.0	0.0	0.0
	8	0.0	0.0	0.0	0.0	0.0	0.0	0.0	0.0	0.0	0.0	0.0	0.0	0.0	0.0	0.0	0.0	0.0	0.0	0.0	0.0	0.0	0.0	0.0	0.0
	9	0.0	0.0	0.0	0.0	0.0	0.0	0.0	0.0	0.0	0.0	0.0	0.0	0.0	0.0	0.0	0.0	0.0	0.0	0.0	0.0	0.0	0.0	0.0	0.0
	10	0.0	0.0	0.0	0.0	0.0	0.0	0.0	0.0	0.0	0.0	0.0	0.0	0.0	0.0	0.0	0.0	0.0	0.0	0.0	0.0	0.0	0.0	0.0	0.0

Supplementary Table 5

Rarefaction	AKGDGQNYFDYSGMDV	AKRASTIAVRLYYFDF	ARVLTASMVRGVIIGNYYYGMDV	AKDSQGWFGNLLNYFDQ	ARDTGSSWYTNWFDP	AREGYEFSMDV
1	1	-	2	3	4	-
2	-	1	3	2	4	-
3	1	-	3	4	-	2
4	-	1	2	3	-	4
5	-	1	3	2	-	4
6	1	-	2	3	-	4
7	-	1	3	2	4	-
8	1	-	2	3	-	4
9	1	-	2	3	4	-
10	-	1	3	-	4	-
	AKGDCQNYFDYSGMDV	AIRASTIAVRLYYFDF	AGVLTASMVRGVIIGNYYYGMDV	AEDSQGWFGNLLNYFDY	AGDTGSSWYTNWFDP	AGEGYEFSMDV
	AKGDGHNYFDYSGMDV	AKMASTIAVRLYYFDF	ARALTASMVRGVIIGNYYYGMDV	AIDSQGWFGNLLNYFDQ	AIDTGSSWYTNWFDP	ARDGYEFSMDV
	AKGDGKNYFDYSGMDV	AKRAATIAVRLYYFDF	ARDLTASMVRGVIIGNYYYGMDV	AIDSQGWFGNLLNYFDY	ARATGSSWYTNWFDP	AREAYDFSMDV
	AKGDGLNYFDYSGMDV	AKRASTIAARLYYFDF	ARFLTASMVRGVIIGNYYYGMDV	AKASQGWFGNLLNYFDQ	ARDAGSSWYTNWFDP	AREDYDFSMDV
	AKGDGQIYFDYSGMDV	AKRASTIAFRLYYFDF	ARVLAASMVRGVIIGNYYYGMDV	AKDFQGWFGNLLNYFDQ	ARDKGSSWYTNWFDP	AREEYDFSMDV
	AKGDGQNFFDYSGMDV	AKRASTIAGRLYYFDF	ARVLTAAMVRGVIIGNYYYGMDV	AKDPQGWFGNLLNYFDQ	ARDTCSSWYTNWFDP	AREEYEFSMDV
	AKGDGQNYFAYSGMDV	AKRASTIAVLLYYFDF	ARVLTASLVRGVIIGNYYYGMDV	AKDSHGWFGNLLNYFDY	ARDTGCSWYTNWFDP	AREGCEFSMDV
	AKGDGQNYFDFSGMDV	AKRASTIAVPLYYFDF	ARVLTASMVLGVIIGNYYYGMDV	AKDSLGWFGNLLNYFDQ	ARDTGGSWYTNWFDP	AREGDDFSLDA
	AKGDGQNYFDYCGMDV	AKRASTIAVRIYYFDF	ARVLTASMVQGVIIGNYYYGMDV	AKDSLGWFGNLLNYFDY	ARDTGISWYTNWFDP	AREGDEFSMDV
	AKGDGQNYFDYSCMDV	AKRASTIAVRLNYFDF	ARVLTASMVRGVIIGDYYYGMDV	AKDSQGCFGNLLNYFDQ	ARDTGSCWYTNWFDP	AREGFDFSLDA
	AKGDGQNYFDYSGIDV	AKRASTIAVRLYNFDF	ARVLTASMVRGVIIGIYYYGMDV	AKDSQGWCGNLLNYFDQ	ARDTGSIWYTNWFDP	AREGNDFSLDA
	AKGDGQNYFDYSGMAV	AKRASTIAVRLYYFDF	ARVLTASMVRGVIIGNCYYGMDV	AKDSQGWFENLLNYFDQ	ARDTGSNWYTNWFDP	AREGYDFSLAA
	AKGDGQNYFDYSGMDA	AKRASTIAVRLYYFDS	ARVLTASMVRGVIIGNYCYGMDV	AKDSQGWFGDLLNYFDQ	ARDTGSRWYTNWFDP	AREGYDFSLDA
	AKGDGQNYFDYSGMDG	AKRASTIAVRLYYFGF	ARVLTASMVRGVIIGNYFYGMDV	AKDSQGWFGILLNYFDY	ARDTGSSWDTNWFDP	AREGYDFSLDS
	AKGDGQNYFDYSGMDV	AKRASTIAVRLYYFVF	ARVLTASMVRGVIIGNYHYGMDV	AKDSQGWFGKLLNYFDQ	ARDTGSSWFTNWFDP	AREGYDFSLVA
	AKGDGQNYFDYSGMVV	AKRASTIAVRLYYFYF	ARVLTASMVRGVIIGNYYNGMDV	AKDSQGWFGNFLNYFDQ	ARDTGSSWNTNWFDP	AREGYEFSLDA
	AKGDGQNYFDYSGMYV	AKRASTIAVRLYYIDF	ARVLTASMVRGVIIGNYYYCMDV	AKDSQGWFGNILNYFDQ	ARDTGSSWYTKWFDP	AREGYEFSMDA
	AKGDGQNYFDYSVMDV	AKRASTIAVRLYYLDF	ARVLTASMVRGVIIGNYYYGIDV	AKDSQGWFGNILNYFDY	ARDTGSSWYTNCFDP	AREGYEFSMDL
	AKGDGQNYFDYYGMDV	AKRASTIAVRQYYFDF	ARVLTASMVRGVIIGNYYYGLDV	AKDSQGWFGNLFNYFDQ	ARDTGSSWYTNWFDL	AREGYEFSMDV
	AKGDGQNYFVYSGMDV	AKRASTIAVSLYYFDF	ARVLTASMVRGVIIGNYYYGMDA	AKDSQGWFGNLINYFDQ	ARDTGSSWYTNWFDP	AREGYEFSMYV
	AKGDGQNYIDYSGMDV	AKRASTIEVRLYYFDF	ARVLTASMVRGVIIGNYYYGMDD	AKDSQGWFGNLLDYFDQ	ARDTGSSWYTNWFDT	AREGYEISMDV
	AKGDGQNYLDYSGMDV	AKRASTISVRLYYFDF	ARVLTASMVRGVIIGNYYYGMDG	AKDSQGWFGNLLKYFDQ	ARDTGSSWYTNWFVP	AREGYELSMDV
	AKGDVQNYFDYSGMDV	AKRASTKAVRLYYFDF	ARVLTASMVRGVIIGNYYYGMDV	AKDSQGWFGNLLNFFDQ	ARDTGSSWYTNWFYP	AREGYEYSMDV
	AKGEGQNYFDYSGMDV	AKRASTMAVRLYYFDF	ARVLTASMVRGVIIGNYYYGMEV	AKDSQGWFGNLLNNFDQ	ARDTGSSWYTSWFDP	AREGYNFSMDV
	AKGNGQNYFDYSGMDV	AKRDSTIAVRLYYFDF	ARVLTASMVRGVIIGNYYYGMGV	AKDSQGWFGNLLNYFAQ	ARDTGSSWYTTWFDP	AREGYVFSLDA
	AKGVGQNYFDYSGMDV	AKRTSTIAVRLYYFDF	ARVLTASMVRGVIIGNYYYGMVV	AKDSQGWFGNLLNYFDE	ARDTVSSWYTNWFDP	ARERYDFSMDV
	AKVDGQNYFDYSGMDV	ANRASTIAVRLYYFDF	ARVLTASMVRGVIIGNYYYGMYV	AKDSQGWFGNLLNYFDQ	ARETGSSWYTNWFDP	ARERYEFSMDV
	ARGDCQNYFDYSGMDV	EKRASTIAVRLYYFDF	ARVLTASMVRGVIIGNYYYGVDV	AKDSQGWFGNLLNYFDR	ARGTGSSWYTNWFDP	ARESYDFSMDV
	ARGDGLNYFDYSGMDV	SKRASTIAVRLYYFDF	ARVLTASMVRGVIIGNYYYSMDV	AKDSQGWFGNLLNYFDS	ARVTGSSWYTNWFDP	AREVYDFSLDA
	ARGDGQNYFDYSGIDV		ARVLTASMVRGVIIGNYYYVMDV	AKDSQGWFGNLLNYFEQ	ARYTGSSWYTNWFDP	AREWYEFSMDV
	ARGDGQNYFDYYGMDV		ARVLTASMVRGVIIGSYYYGMDV	AKDSQGWFGNLLNYFEY	ASDTGSSWYTNWFDP	ARVGYDFSLDA
	ARGDVQNYFDYSGMDV		ARVLTASMVRGVIIGYYYYGMDV	AKDSQGWFGNLLNYFGQ	SRDTGSSWYTNWFDP	ARVGYEFSMDV
	SKGDGQNYFDYSGMDV		ARVLTASMVRGVIIVNYYYGMDV	AKDSQGWFGNLLNYFGY	VRDTGSSWYTNWFDP	ASEGYEFSMDV
			ARVLTASMVRGVIIWNYYYGMDV	AKDSQGWFGNLLNYFVQ		EREGYEFSMDV
			ARVLTASMVRGVINGNYYYGMDV	AKDSQGWFGNLLNYFVY		SREGYEFSMDV
			ARVLTASMVRGVTIGNYYYGMDV	AKDSQGWFGNLLNYFYQ		
			ARVLTASMVRRVIIGNYYYGMDV	AKDSQGWFGNLLNYFYY		
			ARVLTASTVRGVIIGNYYYGMDV	AKDSQGWFGNLLNYIDQ		
			ARVLTSSMVRGVIIGNYYYGMDV	AKDSQGWFGNLLSYFDQ		
			ARVMTASMVRGVIIGNYYYGMDV	AKDSQGWFGSLLNYFDQ		
			ARVQTASMVRGVIIGNYYYGMDV	AKDSQGWFGSLLNYFDY		
			ERVLTASMVRGVIIGNYYYGMDV	AKDSQGWFGYLLNYFDQ		
			SRVLTASMVRGVIIGNYYYGMDV	AKDSQGWFRNLLNYFDQ		
				AKDSQGWFRNLLNYFDY		
				AKDSQGWSGNLLNYFDQ		
				AKDSQGWYGNLLNYFDQ		
				AKDSQRWFGNLLNYFDQ		
				AKDSRGWFGNLLNYFDQ		
				AKDTQGWFGNLLNYFDQ		
				AKDYQGWFGNLLNYFDQ		
				AKESQGWFGNLLNYFDQ		
				AKESQGWFGNLLNYFDY		
				AKGSQGWFGNLLNYFDQ		
				AKYSQGWFGNLLNYFDQ		
				ANDSQGWFGNLLNYFDY		
				EKDSQGWFGNLLNYFDQ		
				PKDSQGWFGNLLNYFDQ		
				VKDSQGWFGNLLNYFDQ		

Supplementary Table 6

	Cluster Rank	1	2	3	4	5	6	7	8	9	10	11	12	13	14	15	16	17	18	19	20	21	22	23	24	25
SVC Coefficients in 10 Rarefaction Datasets	1	0.0	9.4	0.0	0.0	0.0	-3.8	0.0	0.0	0.0	0.0	0.0	0.0	0.0	0.0	12.8	0.0	12.4	0.0	0.0	0.0	0.0	0.0	0.0	0.0	0.0
	2	9.7	0.0	0.0	0.0	0.0	-4.1	0.0	0.0	19.6	0.0	0.0	0.0	0.0	12.6	12.2	0.0	0.0	0.0	0.0	0.0	0.0	0.0	0.0	0.0	0.0
	3	10.5	0.0	0.0	0.0	0.0	0.0	0.0	0.0	-4.5	0.0	0.0	0.0	0.0	0.0	20.5	0.0	0.0	0.0	0.0	0.0	0.0	0.0	0.0	12.2	0.0
	4	0.0	0.0	14.1	0.0	0.0	0.0	0.0	-5.8	0.0	0.0	1.5	17.6	0.0	0.0	0.0	0.0	11.0	0.0	0.0	0.0	0.0	0.0	0.0	9.1	0.0
	5	0.0	0.0	14.1	0.0	0.0	0.0	0.0	0.0	-5.8	0.0	0.0	17.6	0.0	0.0	0.0	0.0	0.0	0.0	0.0	11.1	0.0	0.0	0.0	0.0	0.0
	6	7.7	0.0	0.0	0.0	0.0	0.0	0.0	0.0	-5.6	0.0	19.6	0.0	0.0	0.0	0.0	0.0	0.0	0.0	0.0	0.0	0.0	0.0	11.7	12.4	0.0
	7	8.8	0.0	0.0	0.0	0.0	-6.0	0.0	0.0	0.0	0.0	0.0	0.0	0.0	0.0	0.0	0.0	0.0	0.0	0.0	0.0	0.0	11.7	12.3	0.0	0.0
	8	9.1	0.0	0.0	0.0	0.0	0.0	0.0	0.0	0.0	0.0	0.0	0.0	0.0	0.0	0.0	0.0	0.0	0.0	0.0	0.0	0.0	12.3	0.0	0.0	19.7
	9	9.5	0.0	0.0	0.0	0.0	0.0	0.0	0.0	-5.8	0.0	0.0	0.0	0.0	0.0	0.0	0.0	0.0	0.0	19.8	12.4	0.0	0.0	0.0	0.0	0.0
	10	0.0	14.2	0.0	0.0	0.0	0.0	-5.6	0.0	0.0	0.0	0.0	17.6	0.0	0.0	0.0	0.0	11.1	0.0	0.0	0.0	0.0	0.0	0.0	0.0	10.4
LR Coefficients in 10 Rarefaction Datasets	1	0.0	10.6	8.0	0.0	0.0	-13.0	0.0	0.0	0.0	0.0	0.0	0.0	0.0	0.0	29.4	0.0	28.5	0.0	0.0	0.0	0.0	0.0	0.0	0.0	0.0
	2	17.6	1.6	0.0	0.0	0.0	-13.8	0.0	0.0	42.1	0.0	0.0	-1.9	0.0	29.4	28.4	0.0	0.0	0.0	0.0	0.0	0.0	0.0	0.0	0.0	0.0
	3	13.8	0.0	0.0	0.0	0.0	6.9	-2.0	0.0	-13.9	0.0	0.0	0.0	5.1	0.0	41.2	0.0	0.0	0.0	0.0	0.0	0.0	0.0	0.0	21.9	0.0
	4	0.1	5.9	19.9	0.0	-0.5	0.0	0.0	-14.7	-2.2	0.0	6.9	40.5	0.0	0.0	0.0	0.0	27.6	0.0	0.0	0.0	0.0	0.0	0.0	19.8	0.0
	5	6.6	0.0	18.9	0.0	0.0	0.0	0.0	-2.1	-14.3	0.0	5.7	41.0	0.0	0.0	0.0	0.0	0.0	0.0	0.0	28.0	0.0	0.0	0.0	0.0	0.0
	6	10.9	5.0	0.0	0.0	0.0	0.0	0.0	0.0	-15.4	0.0	42.0	0.0	-1.8	0.0	0.0	0.0	0.0	5.2	0.0	0.0	0.0	0.0	22.3	28.7	0.0
	7	12.8	5.0	0.0	0.0	0.0	-15.9	0.0	0.0	-1.8	0.0	0.0	0.0	0.0	0.0	0.0	0.0	0.0	0.0	0.0	0.0	0.0	27.4	28.7	0.0	0.0
	8	18.4	0.0	0.0	0.0	0.0	-1.5	0.0	0.0	0.0	0.0	0.0	0.0	0.0	0.0	0.0	-15.3	0.0	0.0	0.0	0.0	0.0	29.4	0.0	0.0	43.3
	9	10.6	8.3	0.0	-1.7	0.0	0.0	0.0	0.0	-15.1	0.0	0.0	0.0	0.0	0.0	0.0	0.0	0.0	0.0	42.5	28.8	0.0	0.0	0.0	0.0	0.0
	10	0.0	17.9	7.7	0.0	-2.0	0.0	-14.0	0.0	0.0	0.0	0.0	41.2	2.8	0.0	0.0	0.0	28.1	0.0	0.0	0.0	0.0	0.0	0.0	0.0	24.3

	Cluster Rank	26	27	28	29	30	31	32	33	34	35	36	37	38	39	40	41	42	43	44	45	46	47	48	49	50
SVC Coefficients in 10 Rarefaction Datasets	1	0.0	0.0	0.0	19.7	0.0	0.0	0.0	0.0	0.0	0.0	0.0	0.0	0.0	0.0	0.0	0.0	0.0	0.0	0.0	0.0	0.0	0.0	0.0	0.0	0.0
	2	0.0	0.0	0.0	0.0	0.0	0.0	0.0	0.0	0.0	0.0	0.0	0.0	0.0	0.0	0.0	0.0	0.0	0.0	0.0	0.0	0.0	0.0	0.0	0.0	0.0
	3	0.0	0.0	12.8	0.0	0.0	0.0	0.0	0.0	0.0	0.0	0.0	0.0	0.0	0.0	0.0	0.0	0.0	0.0	0.0	0.0	0.0	0.0	0.0	0.0	0.0
	4	0.0	0.0	0.0	0.0	0.0	0.0	0.0	0.0	0.0	0.0	0.0	0.0	0.0	0.0	0.0	0.0	0.0	0.0	0.0	0.0	0.0	0.0	0.0	0.0	0.0
	5	10.4	0.0	0.0	0.0	0.0	0.0	0.0	0.0	0.0	0.0	0.0	0.0	0.0	0.0	0.0	0.0	0.0	0.0	0.0	0.0	0.0	0.0	0.0	0.0	0.0
	6	0.0	0.0	0.0	0.0	0.0	0.0	0.0	0.0	0.0	0.0	0.0	0.0	0.0	0.0	0.0	0.0	0.0	0.0	0.0	0.0	0.0	0.0	0.0	0.0	0.0
	7	0.0	0.0	0.0	0.0	0.0	0.0	0.0	0.0	0.0	0.0	0.0	0.0	0.0	0.0	0.0	0.0	0.0	0.0	0.0	0.0	0.0	23.8	0.0	0.0	0.0
	8	0.0	0.0	0.0	0.0	0.0	0.0	0.0	0.0	0.0	0.0	0.0	0.0	11.6	0.0	0.0	0.0	0.0	0.0	0.0	0.0	0.0	0.0	0.0	0.0	0.0
	9	0.0	0.0	0.0	0.0	0.0	11.8	0.0	0.0	0.0	0.0	0.0	0.0	0.0	0.0	0.0	0.0	0.0	0.0	0.0	0.0	0.0	0.0	0.0	0.0	0.0
	10	0.0	0.0	0.0	0.0	0.0	0.0	0.0	0.0	0.0	0.0	0.0	0.0	0.0	0.0	0.0	0.0	0.0	0.0	0.0	0.0	0.0	0.0	0.0	0.0	0.0
LR Coefficients in 10 Rarefaction Datasets	1	0.0	0.0	0.0	41.8	0.0	0.0	0.0	0.0	0.0	0.0	0.0	0.0	0.0	0.0	0.0	0.0	0.0	0.0	0.0	0.0	0.0	0.0	0.0	0.0	0.0
	2	0.0	0.0	0.0	0.0	0.0	0.0	0.0	0.0	0.0	0.0	0.0	0.0	0.0	0.0	0.0	0.0	0.0	0.0	0.0	0.0	0.0	0.0	0.0	0.0	0.0
	3	0.0	0.0	28.1	0.0	0.0	0.0	0.0	0.0	0.0	0.0	0.0	0.0	0.0	0.0	0.0	0.0	0.0	0.0	0.0	0.0	0.0	0.0	0.0	0.0	0.0
	4	0.0	0.0	0.0	0.0	0.0	0.0	0.0	0.0	0.0	0.0	0.0	0.0	0.0	0.0	0.0	0.0	0.0	0.0	0.0	0.0	0.0	0.0	0.0	0.0	0.0
	5	21.0	0.0	0.0	0.0	0.0	0.0	0.0	0.0	0.0	0.0	0.0	0.0	0.0	0.0	0.0	0.0	0.0	0.0	0.0	0.0	0.0	0.0	0.0	0.0	0.0
	6	0.0	0.0	0.0	0.0	0.0	0.0	0.0	0.0	0.0	0.0	0.0	0.0	0.0	0.0	0.0	0.0	0.0	0.0	0.0	0.0	0.0	0.0	0.0	0.0	0.0
	7	0.0	0.0	0.0	0.0	0.0	0.0	0.0	0.0	0.0	0.0	0.0	0.0	0.0	0.0	0.0	0.0	0.0	0.0	0.0	0.0	0.0	49.3	0.0	0.0	0.0
	8	0.0	0.0	0.0	0.0	0.0	0.0	0.0	0.0	0.0	0.0	0.0	0.0	28.0	0.0	0.0	0.0	0.0	0.0	0.0	0.0	0.0	0.0	0.0	0.0	0.0
	9	0.0	0.0	0.0	0.0	0.0	27.7	0.0	0.0	0.0	0.0	0.0	0.0	0.0	0.0	0.0	0.0	0.0	0.0	0.0	0.0	0.0	0.0	0.0	0.0	0.0
	10	0.0	0.0	0.0	0.0	0.0	0.0	0.0	0.0	0.0	0.0	0.0	0.0	0.0	0.0	0.0	0.0	0.0	0.0	0.0	0.0	0.0	0.0	0.0	0.0	0.0

	Cluster Rank	51	52	53	54	55	56	57	58	59	60	61
SVC Coefficients in 10 Rarefaction Datasets	1	0.0	0.0	0.0	0.0	0.0	0.0	0.0	0.0	0.0	0.0	0.0
	2	0.0	0.0	0.0	0.0	0.0	0.0	0.0	0.0	0.0	0.0	0.0
	3	0.0	0.0	0.0	0.0	0.0	0.0	0.0	0.0	0.0	0.0	0.0
	4	0.0	0.0	0.0	0.0	0.0	0.0	0.0	0.0	0.0	0.0	0.0
	5	0.0	0.0	0.0	0.0	0.0	0.0	0.0	0.0	0.0	0.0	0.0
	6	0.0	0.0	0.0	0.0	0.0	0.0	0.0	0.0	0.0	0.0	0.0
	7	0.0	0.0	0.0	0.0	0.0	0.0	0.0	0.0	0.0	0.0	0.0
	8	0.0	0.0	0.0	0.0	0.0	0.0	0.0	0.0	0.0	0.0	0.0
	9	0.0	0.0	0.0	0.0	0.0	0.0	0.0	0.0	0.0	0.0	0.0
	10	0.0	0.0	0.0	0.0	0.0	0.0	0.0	0.0	0.0	0.0	0.0
LR Coefficients in 10 Rarefaction Datasets	1	0.0	0.0	0.0	0.0	0.0	-1.9	0.0	0.0	0.0	0.0	0.0
	2	0.0	0.0	0.0	0.0	0.0	0.0	0.0	0.0	0.0	0.0	0.0
	3	0.0	0.0	0.0	0.0	0.0	0.0	0.0	0.0	0.0	0.0	0.0
	4	0.0	0.0	0.0	0.0	0.0	0.0	0.0	0.0	0.0	0.0	0.0
	5	0.0	0.0	0.0	0.0	0.0	0.0	0.0	0.0	0.0	0.0	0.0
	6	0.0	0.0	0.0	0.0	0.0	0.0	0.0	0.0	0.0	0.0	0.0
	7	0.0	0.0	0.0	0.0	0.0	0.0	0.0	0.0	0.0	0.0	0.0
	8	0.0	0.0	0.0	0.0	0.0	0.0	0.0	0.0	0.0	0.0	0.0
	9	0.0	0.0	0.0	0.0	0.0	0.0	0.0	0.0	0.0	0.0	0.0
	10	0.0	0.0	0.0	0.0	0.0	0.0	0.0	0.0	0.0	0.0	0.0

Supplementary Table 7

	ARDNRGLGDS	ARDLVRSSSSFDL	ARWRGMNFWSGYYFDY	VRDRCSTSCYHGEY	ARDIQRTGDY	ASAPYGGYPD	ATGGYSYGLNQY	ARDPYSSGWPVGYYMDV	ATSSGWSY
1	1	2	3	4	-	-	-	-	-
2	-	2	3	4	1	-	-	-	-
3	1	-	2	-	-	3	4	-	-
4	1	-	3	-	-	4	-	2	-
5	1	-	3	-	-	4	-	2	-
6	-	-	2	4	1	3	-	-	-
7	-	-	2	4	-	3	-	-	1
8	1	-	2	4	-	3	-	-	-
9	1	-	2	4	-	3	-	-	-
10	1	-	3	-	-	4	-	2	-
	AGDNRGLGDS	AGDLVRSSSSFDL	AKWRGMNFWSGYYFDY	AGDRCSTSCYHAEY	AGDIHRTGDY	ACAPYGGYPD	AIGGYSYGLNQY	AGDPYSSGWPVGYFMDV	AASSGWSY
	AIDNRGLGDS	ARDFVRSSSSFDL	ARRRGMNFWSGYYFDY	AGDRCSTSCYHGEY	AGDIQRTGDY	AIAPYGGYPD	ASGGYSYGLNQH	AGDPYSSGWPVGYYMDV	ASSSGWSY
	ARANRGLGDS	ARDLGRSSSSFDL	ARWPGMNFWSGYYFDY	AIDRCSTSCYHGEY	AIDIHRTGDY	ANAPYGGYPD	ASGGYSYGLNQY	AIDPYSSGWPLGYFMDV	ATCSGWSY
	ARDDRGLGDS	ARDLVPSSSSFDL	ARWRAMNFWSGYYFDY	ARDRCCTGCYHGEY	AIDIQRTGDY	ASAPCGGYPD	ATCGYSYGLNQY	AIDPYSSGWPVGYFMDV	ATSRGWSY
	ARDHRGLGDS	ARDLVRCSSSFDL	ARWRGLNFWSGYYFDY	ARDRCCTSCYHCEY	AKDIHRTGDY	ASAPHGGYPD	ATDGYSYGLNQY	AIDPYSSGWPVGYYMDV	ATSSGRSY
	ARDNCGLGDS	ARDLVRGSSSFDL	ARWRGMDFWSGYYFDY	ARDRCCTSCYHVEY	ARDIDRTGDY	ASAPYGGNPD	ATGEYSYGLNQY	AKDPYSSGWPLGYFMDV	ATSSGWSY
	ARDNEGLGDN	ARDLVRNSSSFDL	ARWRGMHFWSGYYFDY	ARDRCSISCYHGEY	ARDIERTGDY	ASAPYGGYPA	ATGGCSYGLNHY	ARDAYSSGWPVGYFMDV	ATSSGWYY
	ARDNEGLGDQ	ARDLVRSCSSFDL	ARWRGMNFGSGYYFDY	ARDRCSTACYHGEY	ARDIHLTGDY	ASAPYGGYPD	ATGGCSYGLSHY	ARDLYSSGWPVGYYMDV	TTSSGWSY
	ARDNEGLGDS	ARDLVRSNSSFDL	ARWRGMNFSSGYYFDY	ARDRCSTGCYHGEY	ARDIHRPGDY	ASAPYGGYPE	ATGGGSYGLNHY	ARDPCSSGWPVGYYMDV	
	ARDNEGLGDY	ARDLVRSSSCFDL	ARWRGMNFWCGYYFDY	ARDRCSTSCCHGEY	ARDIHRSGDY	ASAPYGGYPG	ATGGHSYGLNQY	ARDPFSSGWPVGYFMDV	
	ARDNGGLGDS	ARDLVRSSSFFDL	ARWRGMNFWNGYYFDY	ARDRCSTSCFHCEY	ARDIHRTCDY	ASAPYGGYPY	ATGGLSYGLNHY	ARDPNSSGWPVGYYMDV	
	ARDNPGLGDS	ARDLVRSSSSFDL	ARWRGMNFWSAYYFDY	ARDRCSTSCFHVEY	ARDIHRTGDY	ASAPYGRYPD	ATGGLSYGLSHY	ARDPYCSGWPVGYFMDV	
	ARDNRDLGDS	ARDLVRSSSSFEL	ARWRGMNFWSGSYFDY	ARDRCSTSCHHGEY	ARDIHRTGGY	ASAPYGSYPD	ATGGNSYGLNHY	ARDPYCSGWPVGYYMDV	
	ARDNRGLADS	ARDLVRSSSSFNL	ARWRGMNFWSGYCFDY	ARDRCSTSCYHCEY	ARDIHRTGYY	ASAPYSGYPD	ATGGNSYGLNQY	ARDPYGSGWPLGYFMDV	
	ARDNRGLEDS	ARDLVRSSSSSDL	ARWRGMNFWSGYFFDY	ARDRCSTSCYHGEY	ARDIKRTGDY	ASDPYGGYPD	ATGGSSYGLNQY	ARDPYGSGWPVGYFMDV	
	ARDNRGLGDC	ARDLVRSSSSVDL	ARWRGMNFWSGYHFDY	ARDRCSTSCYNGEY	ARDINRTGDY	ASPPYGGYPD	ATGGYGYGLNQY	ARDPYGSGWPVGYYMDV	
	ARDNRGLGDF	ARDLVRSSSSYDL	ARWRGMNFWSGYSFDY	ARDRCSTSFFHGEY	ARDIPRTGDY	ATAPYGGYPD	ATGGYNYGLNQY	ARDPYISGWPLGYFMDV	
	ARDNRGLGDS	ARDLVRSSSYFDL	ARWRGMNFWSGYYFAY	ARDRCSTSRYHGEY	ARDIQLTGDY	ESAPYGGYPD	ATGGYSCGLNQY	ARDPYSCGWPVGYFMDV	
	ARDNRGLGES	ARDLVRSSWSFDL	ARWRGMNFWSGYYFDY	ARDRFSTSCYHGEY	ARDIQRAGDY	GSAPYGGYPD	ATGGYSFGLNQY	ARDPYSCGWPVGYYMDV	
	ARDNRGLGNS	ARDRVRSSSSFDL	ARWRGMNFWSGYYFNY	ARERCSTSCYHGEY	ARDIQRIGDY	PSAPYGGYPD	ATGGYSHGLNQY	ARDPYSIGWPVGYFMDV	
	ARDNRGLGVS	ARGLVRSSSSFDL	ARWRGMNFWSGYYFVY	ARYRCSTSCYHGEY	ARDIQRKGDY	TSAPYGGYPD	ATGGYSSGLNQY	ARDPYSIGWPVGYYMDV	
	ARDNRGLGYS	ARNLVRSSSSFDL	ARWRGMNFWSGYYYDY	ERDRCCTTCYHGEY	ARDIQRPGDY		ATGGYSYGLHQY	ARDPYSSDWPVGYYMDV	
	ARDNRGLRDS	ARYLVRSSSSFDL	ARWRGMNFWTGYYFDY	ERDRCSSSCYHGEH	ARDIQRRGDY		ATGGYSYGLNEH	ARDPYSSGRPLGYFMDV	
	ARDNRGWGDS	GRDLVRSSSSFDL	ARWRGMNSWSGYYFDY	ERDRCSTSCYHAEY	ARDIQRTGDF		ATGGYSYGLNEY	ARDPYSSGRPVGYFMDV	
	ARDNRRLGDS	PRDLVRSSSSFDL	ARWRGMNVWSGYYFDY	GRDRCSTSCYHGEY	ARDIQRTGDH		ATGGYSYGLNHH	ARDPYSSGRPVGYYMDV	
	ARDNRSLGDS	TRDLVRSSSSFDL	ARWRGMNYWSGYYFDY	LRDRCCTTCYHGEY	ARDIQRTGDY		ATGGYSYGLNKH	ARDPYSSGSPVGYFMDV	
	ARDNSGLGDS		ATWRGMNFWSGYYFDY	LRDRCSSSCYHGEH	ARDIQRTGGY		ATGGYSYGLNPY	ARDPYSSGSPVGYYMDV	
	ARDSRGLGDS		GRWRGMNFWSGYYFDY	LRDRCSTSCYHGEH	ARDIQRTGYY		ATGGYSYGLNQC	ARDPYSSGWAVGYFMDV	
	ARDYRGLGDS		PRWRGMNFWSGYYFDY	LRDRCSTSCYHGEY	ARDLHRTGDY		ATGGYSYGLNQD	ARDPYSSGWAVGYYMDV	
	ARENRGLGDS			MRDRCCTTCYHGEY	ARDMHRTGDY		ATGGYSYGLNQQ	ARDPYSSGWHVGYFMDV	
	ARVNRGLGDS			MRDRCSSSCYHGEH	ARDVQRTGDY		ATGGYSYGLNQS	ARDPYSSGWPAGYFMDV	
	ARYNRGLGDS			MRDRCSTSCYHGEY	ARGIQRTGDY		ATGGYSYGLNQY	ARDPYSSGWPAGYYMDV	
	ASDNRGLGDS			SRDRCSTSCYHAEY	ARYIQRTGDY		ATGGYSYGLNRY	ARDPYSSGWPEGYFMDV	
	ATDNRGLGDS			SRDRCSTSCYHGEY	ERDIQRTGDY		ATGGYSYGLYQY	ARDPYSSGWPLGYFIDV	
	DRDNRGLGDS			TRDRCSTSCYHGEY	SRDIHRTGDY		ATGGYSYRLNQH	ARDPYSSGWPLGYFLDV	
	GRDNRGLGDS			VGDRCSTSCYHGEH	SRDIQRTGDY		ATGGYSYWLNQH	ARDPYSSGWPLGYFMEV	
	PRDNRGLGDS			VGDRCSTSCYHGEY	TRDIQRTGDY		ATGGYSYWLNQY	ARDPYSSGWPLGYYMDV	
	SRDNRGLGDS			VMDRCCTTCYHGEY	VRDIQRTGDY		ATGGYTYGLNQY	ARDPYSSGWPLRYFMDV	
	TRDNRGLGDS			VRARCSSSCYHGEH			ATGRYSYGLNQH	ARDPYSSGWPLSYFMDV	
				VRARCSTSCYHGEH			ATGRYSYGLNQY	ARDPYSSGWPLVYFMDV	
				VRARCSTSCYHGEY			ATGVYSYGLNQH	ARDPYSSGWPMGYFMDV	
				VRDGCSSSCYHGEH			ATGVYSYGLNQN	ARDPYSSGWPMGYYMDV	
				VRDLCSSSCYHGEY			ATGVYSYGLNQY	ARDPYSSGWPVCYFMDV	
				VRDLCSTSCFHGEY			ATSGYSYGLNQY	ARDPYSSGWPVCYYMDV	
				VRDLCSTSCYHGEY			GTGGYSYGLNQY	ARDPYSSGWPVGFYMDV	
				VRDQCCTTCYHGEY			PTGGYSYGLNQY	ARDPYSSGWPVGHFMDV	
				VRDQCSSSCYHGEH			STCGNSYGLNQY	ARDPYSSGWPVGHYMDV	
				VRDQCSTSCYHGEY			TAGGYSYGLNQY	ARDPYSSGWPVGYCMDV	
				VRDRCCSSCYHGEH			TPGGYSYGLNQY	ARDPYSSGWPVGYFLDV	
				VRDRCCTACYHGEY			TSGGYSYGLNQH	ARDPYSSGWPVGYFMDA	
				VRDRCCTSCYHGEY			TSGGYSYGLNQY	ARDPYSSGWPVGYFMDF	
				VRDRCCTTCCHGEY			TTCGNSYGLNQY	ARDPYSSGWPVGYFMDV	
				VRDRCCTTCSHGEY			TTCGYSYGLNQY	ARDPYSSGWPVGYFMEV	
				VRDRCCTTCYDGEY			TTGAYSYGLNQY	ARDPYSSGWPVGYFMGV	
				VRDRCCTTCYHGEF			TTGEYSYGLNQY	ARDPYSSGWPVGYFMYV	
				VRDRCCTTCYHGEY			TTGGCSYGLNQY	ARDPYSSGWPVGYHMDV	
				VRDRCCTTCYHGKY			TTGGFSYGLNQY	ARDPYSSGWPVGYSMDV	
				VRDRCCTTCYHGQY			TTGGNSYRLNQY	ARDPYSSGWPVGYYIDV	
				VRDRCCTTCYHGVY			TTGGNSYVLNQY	ARDPYSSGWPVGYYMDA	
				VRDRCCTTCYLGEY			TTGGYGYGLNQY	ARDPYSSGWPVGYYMDF	
				VRDRCCTTCYYGEY			TTGGYNYGLNQY	ARDPYSSGWPVGYYMDG	
				VRDRCCTTFYHGEY			TTGGYSFGLNQY	ARDPYSSGWPVGYYMDV	
				VRDRCGSSCYHGEH			TTGGYSYGLHQY	ARDPYSSGWPVGYYMGV	
				VRDRCGTTCYHGEY			TTGGYSYGLNHH	ARDPYSSGWPVGYYMVV	
				VRDRCITSCYHGEH			TTGGYSYGLNPY	ARDPYSSGWPVGYYMYV	
				VRDRCNSSCYHGEH			TTGGYSYGLNQC	ARDPYSSGWPVGYYVDV	
				VRDRCNTSCYHGEY			TTGGYSYGLNQD	ARDPYSSGWPVRYYMDV	
				VRDRCRTTCYHGEY			TTGGYSYGLNQF	ARDPYSSGWPVSYFMDV	
				VRDRCSASCYHGEY			TTGGYSYGLNQQ	ARDPYSSGWSVGYFMDV	
				VRDRCSGSCYHGEH			TTGGYSYGLNQY	ARDPYSSGWTLGYFMDV	
				VRDRCSPSCYHGEY			TTGGYSYGLNRY	ARDPYSSGWTVGYFMDV	
				VRDRCSRSCYHGEH			TTGGYSYGPNQY	ARDPYSTGWPVGYYMDV	
				VRDRCSSCCYHGEH			TTGGYSYGRNQY	ARDRYSSGWPVGYFMDV	
				VRDRCSSICYHGEH			TTGGYSYRLNQY	ARDRYSSGWPVGYYMDV	
				VRDRCSSSCCHGEH			TTGGYSYVLNQY	ARDSYSSGWPVGYYMDV	
				VRDRCSSSCDHGEH			TTGGYSYWLNQH	AREPYSSGWPLGYFMDV	
				VRDRCSSSCFHGEH			TTGGYSYWLNQY	ARGPYSSGWPLGYFMDV	
				VRDRCSSSCYHAEH			TTGGYTYGLNQY	ARGPYSSGWPVGYFMDV	
				VRDRCSSSCYHDEH			TTGRNSYGLNQY	ARYPYSSGWPVGYFMDV	
				VRDRCSSSCYHGDH			TTGRYSYGLNQH	ARYPYSSGWPVGYYMDV	
				VRDRCSSSCYHGEH			TTGRYSYGLNQY	ASDPYSSGWPVGYFMDV	
				VRDRCSSSCYHGER			TTGVNSYGLNQY	ASDPYSSGWPVGYYMDV	
				VRDRCSSSCYHGQH			TTGVYSYGLNQH	GRDPYSSGWPVGYYMDV	
				VRDRCSSSCYHGVH			TTSGNSYGLNQY	TRDPYSSGWPVGYFMDV	
				VRDRCSSSCYHREH			TTSGYSYGLNQY	VRDPYSSGWPVGYFMDV	
				VRDRCSSSCYHSEH			VTGGYSYGLNQY		
				VRDRCSSSCYHVEH					
				VRDRCSSSCYLGEH					
				VRDRCSSSCYYGEH					
				VRDRCSSSGYHGEH					
				VRDRCSSSSYHGEH					
				VRDRCSSSYYHGEH					
				VRDRCSSTCYHGEH					
				VRDRCSTACYHGEF					
				VRDRCSTACYHGER					
				VRDRCSTACYHGES					
				VRDRCSTGCYHGEC					
				VRDRCSTGCYHGEH					
				VRDRCSTGCYHGEY					
				VRDRCSTICYHGEH					
				VRDRCSTNCYHGEY					
				VRDRCSTRCYHGEH					
				VRDRCSTRCYHGEY					
				VRDRCSTSCDHGEY					
				VRDRCSTSCFHCEY					
				VRDRCSTSCFHGDY					
				VRDRCSTSCFHSDY					
				VRDRCSTSCLHGEY					
				VRDRCSTSCSHGEY					
				VRDRCSTSCYDGEH					
				VRDRCSTSCYDGEY					
				VRDRCSTSCYHGDH					
				VRDRCSTSCYHGDY					
				VRDRCSTSCYHGEH					
				VRDRCSTSCYHGEL					
				VRDRCSTSCYHGEN					
				VRDRCSTSCYHGES					
				VRDRCSTSCYHGEY					
				VRDRCSTSCYHGGY					
				VRDRCSTSCYHGQY					
				VRDRCSTSCYHREY					
				VRDRCSTSCYHVEY					
				VRDRCSTSCYLGEH					
				VRDRCSTSCYLGEY					
				VRDRCSTSCYNGEH					
				VRDRCSTSCYNGEY					
				VRDRCSTSCYQGEH					
				VRDRCSTSCYRGEY					
				VRDRCSTSCYYGEH					
				VRDRCSTSFFHGEY					
				VRDRCSTSFYHGEH					
				VRDRCSTSFYHGEY					
				VRDRCSTSGYHGEY					
				VRDRCSTSRYHGEY					
				VRDRCSTSSYHGEH					
				VRDRCSTSWYHGEY					
				VRDRCSTTCYHGEC					
				VRDRCSTTCYHGEY					
				VRDRCSTVCYHGEC					
				VRDRCTSSCYHGEH					
				VRDRCYTTCYHGEY					
				VRDRFSTSCYHGEH					
				VRDRFSTSCYHGEY					
				VRDRGSSSCYHGEH					
				VRDRRSSSCYHGEH					
				VRDRRSTSCYHGEH					
				VRDRRSTSCYHGEY					
				VRDRSSTSCYHGEH					
				VRDRYSSSCYHGEH					
				VRDRYSTSCYHGEH					
				VRDRYSTSCYHGEY					
				VRDWCSTSCYHGEY					
				VRERCCTTCYHGEY					
				VRGRCCTTCYHGEY					
				VRGRCSSSCYHGEH					
				VRGRCSTSCYHGEH					
				VRHRCCTTCYHGEY					
				VRNRCCTTCYHGEY					
				VRNRCSSSCYHGEH					
				VRVRCSSSCYHGEH					
				VSDRCCTTCYHGEY					
				VSDRCSSSCYHGEH					
				VSDRCSTSCYHGEY					
